# Formation, Signaling and Occurrence of Specialized Pro-Resolving Lipid Mediators—What is the Evidence so far?

**DOI:** 10.3389/fphar.2022.838782

**Published:** 2022-03-02

**Authors:** Nils Helge Schebb, Hartmut Kühn, Astrid S. Kahnt, Katharina M. Rund, Valerie B. O’Donnell, Nicolas Flamand, Marc Peters-Golden, Per-Johan Jakobsson, Karsten H. Weylandt, Nadine Rohwer, Robert C. Murphy, Gerd Geisslinger, Garret A. FitzGerald, Julien Hanson, Claes Dahlgren, Mohamad Wessam Alnouri, Stefan Offermanns, Dieter Steinhilber

**Affiliations:** ^1^ Chair of Food Chemistry, Faculty of Mathematics and Natural Sciences, University of Wuppertal, Wuppertal, Germany; ^2^ Department of Biochemistry, Charité-Universitätsmedizin Berlin, Corporate Member of Freie Universität Berlin and Humboldt-Universität zu Berlin, Berlin, Germany; ^3^ Institute of Pharmaceutical Chemistry, Goethe University Frankfurt, Frankfurt, Germany; ^4^ School of Medicine, Systems Immunity Research Institute, School of Medicine, Cardiff University, Cardiff, United Kingdom; ^5^ Département de Médecine, Faculté de Médecine, Centre de Recherche de l’Institut Universitaire de Cardiologie et de Pneumologie de Québec, Canada Excellence Research Chair on the Microbiome-Endocannabinoidome Axis in Metabolic Health (CERC-MEND), Université Laval, Québec, QC, Canada; ^6^ Division of Pulmonary and Critical Care Medicine, Department of Internal Medicine, University of Michigan Medical School, Ann Arbor, MI, United States; ^7^ Rheumatology Unit, Department of Medicine, Karolinska Institutet, Karolinska University Hospital, Stockholm, Sweden; ^8^ Division of Medicine, Department of Gastroenterology, Metabolism and Oncology, Ruppin General Hospital, Brandenburg Medical School, Neuruppin, Germany; ^9^ Department of Molecular Toxicology, German Institute of Human Nutrition Potsdam-Rehbruecke, Nuthetal, Germany; ^10^ Department of Pharmacology, University of Colorado-Denver, Aurora, CO, United States; ^11^ Institute of Clinical Pharmacology, Pharmazentrum Frankfurt, University Hospital of Goethe-University, Frankfurt, Germany; ^12^ Fraunhofer Institute for Translational Medicine and Pharmacology, ITMP and Fraunhofer Cluster of Excellence for Immune Mediated Diseases, CIMD, Frankfurt, Germany; ^13^ Institute for Translational Medicine and Therapeutics, Perelman School of Medicine, University of Pennsylvania, Philadelphia, PA, United States; ^14^ Laboratory of Molecular Pharmacology, GIGA-Molecular Biology of Diseases, University of Liège, Liège, Belgium; ^15^ Laboratory of Medicinal Chemistry, Centre for Interdisciplinary Research on Medicines (CIRM), University of Liège, Liège, Belgium; ^16^ Department of Rheumatology and Inflammation Research, Institute of Medicine, Sahlgrenska Academy, University of Gothenburg, Gothenburg, Sweden; ^17^ Department of Pharmacology, Max Planck Institute for Heart and Lung Research, Bad Nauheim, Germany; ^18^ Center for Molecular Medicine, Goethe University Frankfurt, Frankfurt, Germany

**Keywords:** lipoxygenase, SPM, lipoxin, resolvin, resolution of inflammation, leukotriene, FPR, LC-MS-based lipid mediator analysis

## Abstract

Formation of specialized pro-resolving lipid mediators (SPMs) such as lipoxins or resolvins usually involves arachidonic acid 5-lipoxygenase (5-LO, ALOX5) and different types of arachidonic acid 12- and 15-lipoxygenating paralogues (15-LO1, ALOX15; 15-LO2, ALOX15B; 12-LO, ALOX12). Typically, SPMs are thought to be formed via consecutive steps of oxidation of polyenoic fatty acids such as arachidonic acid, eicosapentaenoic acid or docosahexaenoic acid. One hallmark of SPM formation is that reported levels of these lipid mediators are much lower than typical pro-inflammatory mediators including the monohydroxylated fatty acid derivatives (e.g., 5-HETE), leukotrienes or certain cyclooxygenase-derived prostaglandins. Thus, reliable detection and quantification of these metabolites is challenging. This paper is aimed at critically evaluating i) the proposed biosynthetic pathways of SPM formation, ii) the current knowledge on SPM receptors and their signaling cascades and iii) the analytical methods used to quantify these pro-resolving mediators in the context of their instability and their low concentrations. Based on current literature it can be concluded that i) there is at most, a low biosynthetic capacity for SPMs in human leukocytes. ii) The identity and the signaling of the proposed G-protein-coupled SPM receptors have not been supported by studies in knock-out mice and remain to be validated. iii) In humans, SPM levels were neither related to dietary supplementation with their ω-3 polyunsaturated fatty acid precursors nor were they formed during the resolution phase of an evoked inflammatory response. iv) The reported low SPM levels cannot be reliably quantified by means of the most commonly reported methodology. Overall, these questions regarding formation, signaling and occurrence of SPMs challenge their role as endogenous mediators of the resolution of inflammation.

## *In Vitro* Formation of Lipoxins and Resolvins

In contrast to originally identified oxylipins such as leukotrienes that are conventionally implicated in inflammatory responses, specialized pro-resolving mediators (SPM) represent an alternative group that have been suggested to be important for inhibition of inflammation and promotion of its resolution.

SPMs such as lipoxins and resolvins were reported to foster resolution of inflammation. For example, resolvin D2 (RvD2) was reported to be a potent regulator of leukocyte function and to control microbial sepsis at very low concentrations in mice (Spite et al., 2009). Resolvin E2 (RvE2) was shown to inhibit zymosan-induced leukocyte infiltration and peritoneal inflammation (Tjonahen et al., 2006). Later on, a plethora of biological and pharmacological activities of SPMs were reported such as the limitation of PMN tissue infiltration, enhancement of macrophage phagocytosis and efferocytosis, increased microbial killing and enhanced tissue regeneration [for review see (Serhan, 2017b; Serhan and Levy, 2018)]. The relevance of these findings to the physiological and pathophysiological roles of endogenous SPMs depends on their *in vivo* concentrations and the accuracy with which they can be measured in body fluids.

**FIGURE 1:**
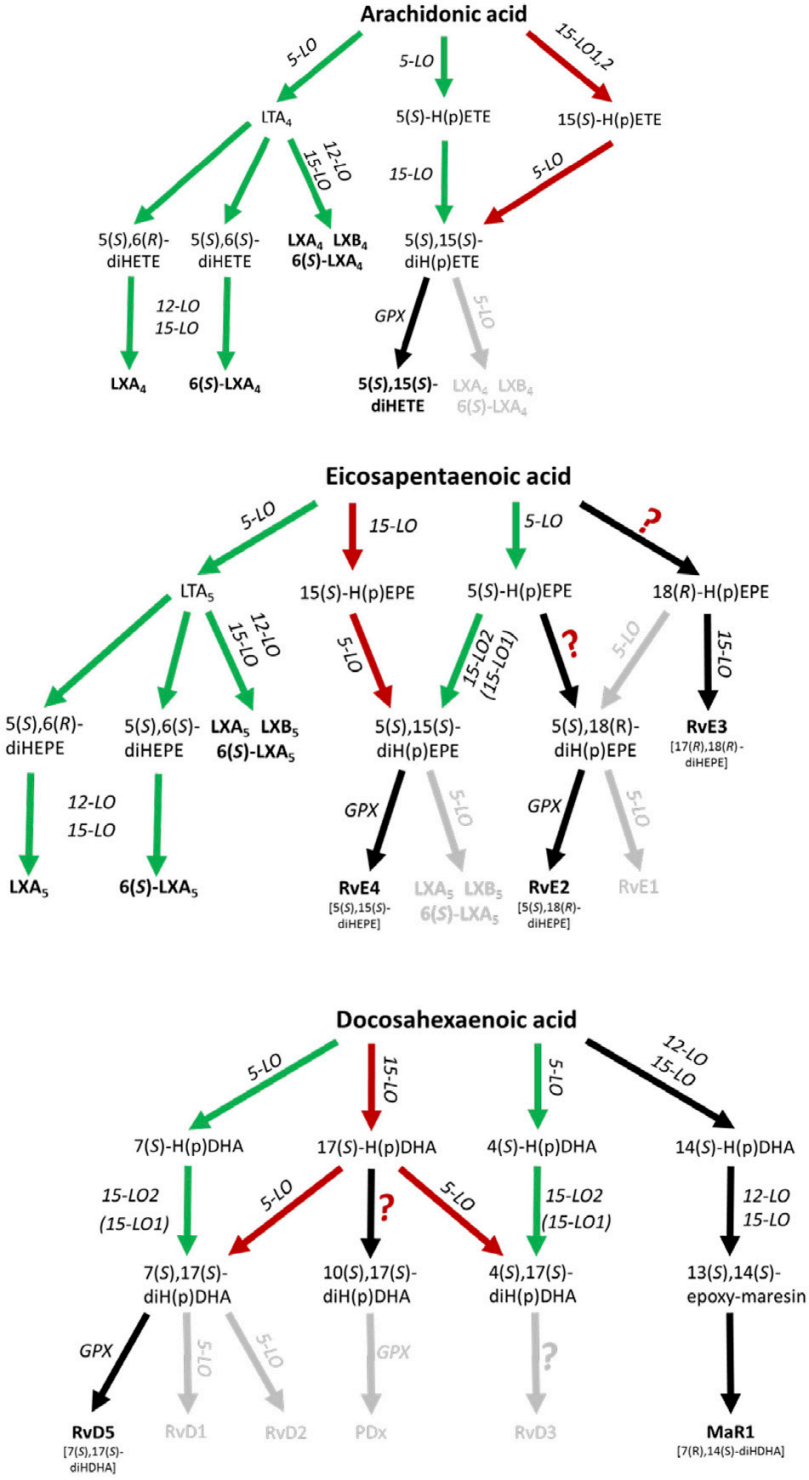
Scheme of human leukocyte-dependent SPM formation from AA, EPA and DHA where SPMs with significant formation are highlighted. The 5-LO:12/15-LO pathway is shown in green and the 12/15-LO:5-LO pathway is depicted in red. Inefficient SPM biosynthesis routes are grey colored. GPX, glutathione peroxidase.

The biosynthetic pathways for lipoxins and resolvins have been suggested to involve at least two consecutive oxygenase reactions. Formation of lipoxins can occur via the oxygenation of arachidonic acid (AA) by 5-lipoxygenase (5-LO, ALOX5) to LTA_4_ followed by further conversion of this intermediate by murine 12/15-LO (Alox15), human 15-LOs (ALOX15, ALOX15B) or 12-LO (ALOX12) to LXA_4_ and LXB_4_, further referred to as the 5-LO:12/15-LO pathway or via the consecutive action of 12/15-LO and 5-LO which is referred to as the 12/15-LO:5-LO pathway (Figure 1). Both SPM biosynthesis pathways can involve transcellular cooperation in which the first oxygenation product is transferred to a neighboring cell type for the second oxygenation. This will be discussed below in more detail. It should also be mentioned here that the mouse orthologue (12/15-LO, Alox15) of the human 15-LO (ALOX15) generates a different product pattern (see *The 12/15-LO:5-LO Pathway of Lipoxin, D-Series Resolvin and RvE4 Formation* and *5-LO-Independent Lipoxin Formation*).

In view of the findings reviewed here, there are significant differences in the biosynthetic capacities for SPM formation. Recent data are compatible with the hypothesis that trihydroxylated SPMs are formed at much lower rates than dihydroxylated SPMs such as resolvin D5, D6, E2 and E4.

### The 5-LO:12/15-LO Pathway of SPM Formation

Here, 5-LO first generates 5-HpETE, 5-HpEPE and 7-HpDHA from AA, EPA and DHA, respectively, and these metabolites are further converted by AA 15-lipoxygenating enzymes (Figure 1). Indeed, it has been shown that isolated 15-LO2 (ALOX15B) equally accepts 5-HETE and 5-HpETE for the formation of 5,15-diHETE (Green et al., 2016). Furthermore, the enzyme also accepts 7-HDHA and 7-HpDHA for conversion to RvD5 (7,17-diHDHA). Human 15-LO1 (ALOX15) also accepts 7-H(p)DHA as substrate but 7,14-diH(p)DHA was identified as major (90%) reaction product (Perry et al., 2020a). These data are compatible with RvD5 formation via the 5-LO:15-LO pathway being 5-LO:15-LO2 restricted.

Efficient transcellular lipoxin formation was detected in platelet/leukocyte co-incubations stimulated with Ca^2+^ ionophore or a combination of fMLF and thrombin. 5-LO-derived LTA_4_ is released from granulocytes and then further converted by platelet 12-LO (ALOX12) to lipoxins (Serhan and Sheppard, 1990; Edenius et al., 1991; Lehmann et al., 2015) (Figure 1). Furthermore, it was shown that LTA_4_ can serve as a substrate for various ALOX15 orthologues (Tornhamre et al., 2000). Thus, in principle, 12-LO and 15-LO are functionally redundant in the biosynthetic cascade.

In contrast to granulocyte (PMNL)/platelet co-incubations, co-expression of 5-LO together with 15-LO1 or 15-LO2 in the same cell type did not result in efficient lipoxin formation *via* the 5-LO:12/15-LO pathway. Human macrophages differentiated in the presence of IL-4 co-express ALOX5 and ALOX15 (Wuest et al., 2012; Ebert et al., 2020; Von Hegedus et al., 2020) and should be able to form lipoxins on their own. However, release of lipoxins upon stimulation with Ca^2+^ ionophore in presence of exogenous AA was barely detectable in these cells (Ebert et al., 2020). At present, the reason for the lack of SPM formation under these experimental conditions is unknown.

Freshly isolated PMNL suspensions contain eosinophils which are known to co-express ALOX5 and ALOX15 (Nadel et al., 1991; Archambault et al., 2018) yet produce only minute amounts of lipoxins, compared to other lipoxygenase metabolites, even when stimulated with Ca^2+^ ionophore (Serhan et al., 1987; Edenius et al., 1991; Mainka et al., 2021). It is not known whether this is due to the fact that PMNL are mainly composed of neutrophils which mainly express ALOX5, while ALOX15 expression is more or less restricted to eosinophils which represent only a minor fraction of the PMNLs. Furthermore, ALOX15B expression was recently demonstrated in neutrophils (Archambault et al., 2018) whereas another study failed to detect this enzyme in granulocytes (Mainka et al., 2021).

Of note, leukocyte preparations are usually contaminated with platelets since these cells tend to adhere to leukocytes during isolation. Thus, contamination with platelet-derived 12-LO cannot be avoided. Therefore, the small amounts of lipoxins occasionally observed in PMNL and monocyte/macrophage preparations might in part be related to platelet contamination.

### The 12/15-LO:5-LO Pathway of Lipoxin, D-Series Resolvin and RvE4 Formation

As an alternative to the 5-LO:12/15-LO pathway, 5-LO together with 15-LO1 (ALOX15) or 15-LO2 (ALOX15B) may produce lipoxins, D-series resolvins and RvE4 from their corresponding PUFAs (Figure 1). In a first step AA, eicosapentaenoic acid (EPA) or docosahexaenoic acid (DHA) can be oxidized to 15-H(p)ETE, 15-H(p)EPE and 17-H(p)DHA, respectively by an AA 15-lipoxygenating enzyme. 15-H(p)ETE on one hand and 15-H(p)EPE on the other are then further converted by 5-LO to 5,15-diHETE, LXA_4_, LXB_4_ and RvE4 (5,15-diHEPE), LXA_5_ and LXB_5_. The formation of SPMs from DHA is more complex and 17-H(p)DHA is reported to be converted to RvD1-6 (Chiang and Serhan, 2017; Serhan and Levy, 2018). With DHA, human 15-LO1 exhibits a pronounced dual specificity since DHA is oxygenated to a 1:1 mixture of 17-HDHA and 14-HDHA (Kutzner et al., 2017) (Figure 1). In contrast, mouse Alox15 forms 100% 14-HDHA (Kutzner et al., 2017). These differences in the reaction specificity between mouse and human ALOX15 orthologues may impact the SPM synthase activities of the two ALOX15 orthologues from different substrates, although this is not known.

A hallmark of the proposed 12/15-LO:5-LO SPM formation route is that the substrate fatty acids (AA, EPA, DHA) are first oxygenated by 15-LO1/15-LO2 or other oxygenases to the corresponding monohydro(pero)xy-fatty acids (15-H(p)ETE, 15-H(p)EPE, 17-H(p)DHA) and subsequently by 5-LO (Figure 1). It should be stressed at this point that the substrates for the second (5-LO-catalyzed) oxygenation reaction (15-H(p)ETE, 15-H(p)EPE, 17-H(p)DHA) may also originate from fatty acid auto-oxidation. If one follows the proposed mechanistic scenario of the 12/15-LO:5-LO pathway there are two major prerequisites for SPM formation:

i) *Involvement of leukocytes*. ALOX5 expression is largely restricted to these immune cells, as red blood cells and platelets are 5-LO deficient (Rådmark et al., 2007).
ii) *5-LO must accept 15-H(p)ETE, 15-H(p)EPE and/or 17-H(p)DHA as substrates for SPM formation*. Interestingly, as will be discussed in *Lipoxin and Resolvin Formation in Human PMNL* and *Lipoxin and Resolvin Formation in Human Macrophages*, the conversion rate of most of these oxylipins is dependent on FLAP, which is normally present in 5-LO-containing cells. In the absence of FLAP, the conversion rate of these oxylipins by purified human 5-LO is very low compared to AA or EPA. In fact, the conversion rate of 15-HpETE to 5,15-diHpETE by human 5-LO was found to be 10-fold lower compared to the conversion of AA to 5-HpETE. Moreover, no conversion of 5,15-diHpETE to LXA_4_ was observed (Green et al., 2018). In line with this finding, 15-HETE is hardly converted to LXA_4_ isomers by purified human 5-LO (Lehmann et al., 2015; Mainka et al., 2021). In fact, compared with AA, conversion of 15-HETE and 15-HpETE by 5-LO was found to be 130- and 85-fold slower, respectively (Perry et al., 2020a). Similarly, the oxygenation rates of 17-HDHA and 17-HpDHA by human 5-LO are more than 100-fold lower when compared with the parent fatty acid DHA (Perry et al., 2020b). In accordance, 17-HDHA was not accepted as substrate by recombinant human 5-LO and 17-HDHA was not converted to resolvins such as RvD5 (Mainka et al., 2021). These data clearly show that in the tested *in vitro* systems in the absence of FLAP, oxylipins are poor substrates for purified human 5-LO. Furthermore, there seem to be no significant differences between the turnover of the hydroxy fatty acids compared to their corresponding hydroperoxy derivatives (Mainka et al., 2021).

### Formation of EPA-Derived 18-HEPE and of the Resolvin E-Series

In a recent publication, the patterns of oxygenation products of EPA and 18-HEPE were analyzed with particular emphasis on the formation of double and triple oxygenation products (Kutzner et al., 2020) (Table 1). It was found that recombinant 5-LO as well as a combination of 5-LO with 15-LO1 oxygenated these substrates to a complex mixture of mono-, double- and triple oxygenation products. Recombinant 5-LO and ALOX15 paralogues formed different double oxygenation products (Table 1) including 5,15-diHEPE and various 8,15-diHEPE isomers (Figure 1). Similar product patterns were identified when the two enzymes were simultaneously added to the assay system. Of note, formation of triple oxygenation products, i.e., RvE1 (Table 1), which has an 18R hydroxyl group was below the detection limits of the employed LC-MS method (see *Quantitative Analysis and SPM Levels in Humans*). When 18(R,S)-HEPE was used as substrate, a number of dihydroxy derivatives including 5,18-diHEPE (RvE2) and 17,18-diHEPE (RvE3) but also small amounts of trihydroxy compounds such as 5,17,18-triHEPE were detected (Kutzner et al., 2020). Thus, it has to be considered whether 18-HEPE is likely to be present as a potential substrate for this *in vivo*. While 18R-HEPE is a precursor in the biosynthesis of different resolvins (RvE1, RvE2, RvE3) (Figure 1), the origin of 18R-HEPE in biological systems remains speculative. LO isoforms have been suggested as a metabolic source but considering the reaction specificity of mammalian LO-isoforms, effective formation of 18R-HEPE from EPA is not very likely. When EPA was incubated with recombinant human 5-LO, 12-LO, 15-LO2 and 15-LO1, very small amounts of 18-HEPE were formed in the 15-LO1 incubations and its *S/R*-ratio was less than 3:1. However, this compound which was hardly detected in the incubation mixtures of the other LO-isoforms, only contributed 1% to the mixture of all oxygenation products (Kutzner et al., 2020). These data indicate that 18-HEPE formation from EPA via the 15-LO pathway is very inefficient and that the stereochemistry of 18-HEPE oxygenation is not tightly controlled by the enzyme. The molecular basis for the low degree of enantio-control has not been explored in detail but it might be speculated that hydrogen abstraction from C(16) is catalyzed by the enzymes whereas oxygen insertion at C(18) proceeds in part non-enzymatically.

**TABLE 1:** Di- and trihydroxylated oxylipins derived from AA, EPA and DHA.

Hydroxylation	Trivial name	Hydroxylation pattern	Proposed pathway of formation
dihydroxy derivatives	5,15-diHETE	5,15-diHETE^a^	12/15-LO:5-LO, 5-LO:12/15-LO
	8,15-diHETE	8,15-diHETE	12/15-LO
	resolvin D5	7,17-diHDHA^b^	12/15-LO:5-LO
	resolvin D6	4,17-diHDHA	12/15-LO:5-LO
	resolvin E2	5,18-diHEPE^c^	CYP or (12/15LO):5-LO
	resolvin E3	17,18-diHEPE	CYP, (12/15-LO)
	resolvin E4	5,15-diHEPE	12/15-LO:5-LO, 5-LO:12/15-LO
trihydroxy derivatives	lipoxin A_4_	5,6,15-triHETE	5-LO:12/15-LO
	lipoxin B_4_	5,14,15-triHETE	5-LO:12/15-LO
	resolvin D1	7,8,17-triHDHA	12/15-LO:5-LO
	resolvin D2	7,16,17-triHDHA	12/15-LO:5-LO
	resolvin D3	4,11,17-triHDHA	12/15-LO:5-LO
	resolvin D4	4,5,17-triHDHA	12/15-LO:5-LO
	resolvin E1	5,12,18-triHEPE	CYP or (12/15-LO):5-LO

a Hydroxyeicosatetraenoic acid.

b Hydroxydocosahexaenoic acid.

c Hydroxyeicosapentaenoic acid.

Alternatively, 18R-HEPE might arise from oxygenation of EPA by CYP450 enzymes (Isobe et al., 2018), but there is no evidence for the physiological relevance of this biosynthetic pathway so far. In this respect, it is of interest that CYP4F18 oxidizes LTB_4_ to 18-OH-LTB_4_ (Christmas et al., 2006) which is a structural analogue to the EPA-derived RvE1 (chemically a 18-OH-LTB_5_). However, it is not known whether LTB_5_ is also a CYP4F18 substrate.

Formation of 15R-HETE from AA by COX2 in stimulated endothelial cells has been reported after COX2 acetylation by aspirin, leading to the so called aspirin-triggered (AT) lipoxins which represent C(15) epimers (Claria and Serhan, 1995). Later it was shown that aspirin treatment of endothelial cells can generate 18R-HEPE from EPA (Serhan et al., 2000). However, in a subsequent publication, it was reported that aspirin treatment of human recombinant COX2 fosters the formation of 18S-HEPE but not the 18R configuration (Oh et al., 2011). In agreement with this finding, slightly enhanced 18S-HEPE formation was found in serum from aspirin-treated patients (Oh et al., 2011). At present, it is unclear whether 18R-HETE is formed *in vivo* to serve as precursor for SPM formation. Thus, we doubt that aspirin-triggered 18-HEPE formation significantly contributes to the resolvin E formation. All of these observations depend on aspirin treatment *in vitro*. Their relevance to the pharmacological actions of aspirin *in vivo* remain entirely speculative.

### 5-LO-Independent Lipoxin Formation

It has been reported 40 years ago that soybean LOX1 is capable of oxygenating its own AA oxygenation product (15-HpETE) to a mixture of several dihydroxy eicosanoids and the major products of this AA double oxygenation were identified as 8S,15S-diHpETE and 5S,15S-diHpETE (Van Os et al., 1981). The reaction rate of the second oxygenation step (conversion of 15S-HpETE to the mixture of 8S,15S-diHpETE and 5S,15S-diHpETE) was about 5-fold lower than the rate of AA oxygenation (Van Os et al., 1981). As a mechanistic reason for C(5) oxygenation of arachidonic acid derivatives, an inverse head-to-tail orientation of the fatty acid substrate at the active site of the enzyme has been suggested. Free polyenoic fatty acids penetrate the active site of AA 15-lipoxygenating ALOX-isoforms leading with their methyl end and this allows optimal alignment of the substrate for C(15) lipoxygenation. In 15-H(p)ETE, the presence of the hydro(pero)xy group in close proximity to the methyl end of the fatty acid substrate reduces the hydrophobicity of this part of the substrate molecule and thus, the substrate might be inversely aligned at the active site. This inverse substrate alignment favors oxygenation at C(5) and C(8). Similar experiments were carried out with pure rabbit 15-LO. When this enzyme was incubated with AA at 37°C significant amounts of 8,15-diHpETE, 5,15-diHpETE as well as 14,15-LTA_4_ were detected as reaction products (Bryant et al., 1985). These data are compatible with AA 15-lipoxygenating LO isoforms capable of catalyzing the oxygenation of other carbon atoms of polyenoic fatty acids when oxygenation of the preferred carbon atom is blocked. 5S,15S-diH(p)ETE may be considered as an intermediate in the formation of lipoxin isomers (5,6,15-triHETE is LXA_4_ and 5,14,15-triHETE is LXB_4_). In fact, when pure rabbit ALOX15 was incubated with 15-HETE as substrate, significant amounts of LXB_4_ (5,14,15-triHETE) were formed (Kühn et al., 1984). The structure of this compound was characterized comprehensively (Kühn et al., 1987) and experiments with heavy oxygen isotopes indicated that the oxygen introduced at carbons 5 and 14 originated from atmospheric ^18^O_2_. These data indicate that LXB_4_ can be formed via 5-LO-independent pathways and that the substrate (15-HETE derivatives) is metabolized via two consecutive steps of 15-LO catalyzed oxygenation. It should, however, be emphasized that in principle, the formation of 5,14,15-triHETE (LXB_4_) may also proceed via an alternative mechanism. For instance, the 14,15-LTA_4_ synthase activity of rabbit 15-LO (Bryant et al., 1985) may lead to the conversion of 15-HpETE to 14,15-LTA_4_. This metabolite quickly undergoes non-enzymatic hydrolysis yielding various 14,15-diHETE isomers. When these isomers undergo 5-lipoxygenation either by 5-LO or by the 5-lipoxygenating activity of 15-LO, 5,14,15-triHETE is formed.

The biosynthetic capacity of human and mouse ALOX-isoforms for lipoxin isomer formation depends on the reaction specificity of these enzymes with arachidonic acid. Human 15-LO1 is an AA 15-lipoxygenating enzyme (Sigal et al., 1990) while its mouse orthologue is dominantly AA 12-lipoxygenating (Sun and Funk, 1996). It may therefore not be possible to conclude the efficiency of lipoxin synthase activity of mouse 12/15-LO (Alox15) from the activity of the human orthologue. In other words, a biosynthetic scheme worked out for humans might not adequately mirror the situation in mice. A similar situation exists for mouse and human ALOX15B. Here, the human enzyme is AA 15-lipoxygenating (Jisaka et al., 2000) whereas mouse Alox15B converts AA mainly to 8-HpETE (Krieg et al., 1998).

Taken together, most of these studies on lipoxin biosynthesis were performed with purified enzymes and under incubation conditions which do not necessarily reflect the cellular environment. Considering the data from intact cell incubations and the FLAP dependency of lipoxin formation, it is rather unlikely that 5-LO-independent pathways are a plausible route to SPM formation *in-vivo*.

### Lipoxin and Resolvin Formation in Human PMNL

Formation of lipoxins by 5-LO was first reported in human leukocytes (Serhan et al., 1984b; a). Although the oxygenation of 15-HETE to 5,15-diHETE by purified human 5-LO is two orders of magnitude less effective than AA oxygenation (Perry et al., 2020a), the enzyme accepts 15-HETE as substrate in a cellular setup where 5-LO interacts with FLAP which transfers the fatty acid substrates to 5-LO (Dixon et al., 1990). In fact, in intact PMNL, 5-LO oxygenates 12- and 15-HETE in a FLAP-dependent manner (Hill et al., 1992; Mancini et al., 1998) (Figure 1). Subsequently, it was found that FLAP is also required for the biosynthesis of LXA_4_ and RvD1 from 15-HETE and 17-HDHA, respectively (Lehmann et al., 2015). Similarly, formation of 5,15-diHETE, RvD1, RvD5 and RvE1 is FLAP-dependent since their formation can be efficiently inhibited by the FLAP inhibitor MK-886 (Mainka et al., 2021). This infers that the investigation of SPM formation with purified enzymes might not adequately mirror the biosynthetic capacity of cellular systems where additional protein-protein and protein-lipid interactions occur. Further investigation of SPM formation by 5-LO was performed in ionophore-stimulated PMNL from AA, DHA as well as the SPM precursors 15-HETE, 17-HDHA and 18-HEPE (Figure 1). Due to the low cellular concentrations of 15-LO1, 15-LO2 and 12-LO in PMNL, no substantial amounts of AA- and DHA-derived SPMs were detected (Mainka et al., 2021). In contrast, human and mouse eosinophils which express ALOX15 biosynthesized docosanoids (14-HDHA, 17-HDHA) in the presence of DHA, but the resolvin levels were very low: RvD5 was the most prominent of these (Archambault et al., 2022). Noteworthy, the stimulation of eosinophils with platelet-activating factor (which increases intracellular Ca^2+^ concentrations and thus 5-lipoxygenase activity) did not increase docosanoids despite increasing leukotrienes.

When 15-HETE was used as a substrate for human PMNL, LXA_4_ and 6S-LXA_4_ formation was about 75- and 110-times lower compared to 5,15-diHETE, suggesting that formation of the 5,6-epoxy-15-hydroxy intermediate is unfavorable (Mainka et al., 2021). Similarly, only the dihydroxylated SPM RvD5 was formed from 17-DHDA in easily detectable amounts. 18-HEPE was barely converted by 5-LO in the PMNL incubations and the formed amounts of RvE1 and RvE2 were more than 30- and 90-fold lower as compared to 17-HDHA-derived RvD5 (Mainka et al., 2021). In contrast to RvE1, detectable RvE2 formation was observed in ionophore-stimulated human macrophages in the presence of EPA as substrate (Ebert et al., 2020). In line with this, it was found that recombinant human 5-LO can catalyze the conversion of 18-HEPE to RvE2 (Kutzner et al., 2020) (Figure 1).

Taken together, all these data suggest that the 12/15-LO:5-LO pathway where the granulocyte 5-LO employs SPM precursors released e.g. from 15-LO1, 12-LO or 15-LO2 expressing cells is an inefficient source of trihydroxy SPMs (lipoxins, resolvins D1-4, resolvin E1) (Figure 1; Table 1). In contrast, the formation of dihydroxy SPMs, such as RvD5 (7,17-diHDHA), RvE2 (5,18-diHEPE), and RvE4 (5,15-diHEPE) appears to be more efficient (Lam et al., 1987), at least if DHA, EPA or the respective oxylipin precursors are added and the cells are stimulated with ionophore. The low formation of trihydroxylated SPMs, even in these *in-vitro* systems, could be related to the fact that formation of the 5,6-epoxide from hydroperoxy precursors by 5-LO is rather slow and that the 5-hydroperoxy intermediates generated by this enzyme are rapidly reduced by glutathione peroxidases to the corresponding alcohols. This peroxide reduction prevents epoxide formation via the leukotriene A_4_ synthase activity of 5-LO.

A major prerequisite for the formation of 5-LO-derived products is the Ca^2+^-dependent translocation of the enzyme to nuclear membranes where it interacts with FLAP. Indeed, a number of stimuli capable of elevating the cytosolic Ca^2+^ concentration are frequently used to obtain detectable SPM formation in human leukocytes. Ca^2+^ ionophore triggers lipoxin (Serhan et al., 1986a; Serhan et al., 1986b; Serhan et al., 1987; Serhan, 1989; Edenius et al., 1991; Sheppard et al., 1992; Chavis et al., 1996; Lehmann et al., 2015; Ebert et al., 2020; Mainka et al., 2021) as well as D-series (Spite et al., 2009; Lehmann et al., 2015; Ebert et al., 2020) and E-series (Tjonahen et al., 2006; Isobe et al., 2012; Ebert et al., 2020) resolvin formation by neutrophils and macrophages. Furthermore, stimuli that activate Gq- and Gi-coupled receptors such as thrombin and fMLF were reported to trigger SPM biosynthesis (Serhan and Sheppard, 1990; Sheppard et al., 1992). Resolvin formation was proposed to be induced by yeast-derived zymosan (Tjonahen et al., 2006; Sun et al., 2007; Spite et al., 2009) and under cell stress conditions such as hypoxia (Norris et al., 2019) and apoptosis (Dalli and Serhan, 2012) or upon co-incubation of macrophages with apoptotic PMNL (Dalli and Serhan, 2012). All these stimuli are also known to mobilize Ca^2+^ from intracellular stores. Furthermore, live/growing bacteria (*E. coli, Staphylococcus aureus*) or bacterial toxins were shown to stimulate SPM biosynthesis in human macrophages (Werz et al., 2018; Rao et al., 2019; Jordan et al., 2020). In these studies, prolonged incubation times of 90–180 min were used.

In recent years, short term incubations (up to 15 min) employing stimuli such as Ca^2+^ ionophore have been shown to produce low levels of SPMs. The SPM formation capacity of different 5-LO stimuli (LPS/fMLF, S1P, phenol soluble modulin-α, Ca^2+^ ionophore and osmotic stress) was recently compared in human PMNL supplemented with 15-HETE and 17-HDHA in short term incubations. These experiments showed that the SPM synthesizing capacity differed markedly depending on the stimulus. Nevertheless, Ca^2+^ ionophore was the most potent stimulus, probably due to the strong intracellular Ca^2+^ mobilization triggered by this compound. The pattern of SPM formation (high levels of dihydroxylated and minute amounts of trihydroxylated lipids) was comparable for all stimuli tested (Mainka et al., 2021) and did not substantially differ from other studies. Longer incubation times were not tested since 5-LO is rapidly auto-inactivated after stimulation (Aharony et al., 1987; Rouzer and Kargman, 1988; Wong et al., 1988; Kargman and Rouzer, 1989). Moreover, during long-term incubations, the probability of nonspecific autoxidation is strongly increased. This clearly argues for the use of Ca^2+^ ionophore in investigations relating to SPM biosynthetic capacity since this non-physiological stimulus is potent enough to trigger SPM biosynthesis in amounts sufficient for LC-MS detection (*Quantitative Analysis and SPM Levels in Humans*) with SPM patterns comparable to other stimuli.

However, it should be emphasized that these conditions (stimulation of leukocytes with Ca^2+^ ionophore, addition of AA, EPA and DHA) do not reflect the *in vivo* situation where weaker cell stimulation and lower substrate availability occurs. Furthermore, in leukocytes, AA is present and released at much higher levels than EPA or DHA from cell membranes upon cell stimulation. This favors formation of AA-derived oxylipins rather than EPA or DHA derived oxylipins under physiological conditions (Lee et al., 1985; Pratt et al., 2002). However, a ω-3 PUFA rich diet or ω-3 PUFA supplementation strongly increases their content in the membrane and several enzymes of the AA cascade including LOs (Kutzner et al., 2017) and CYPs (Fischer et al., 2014) preferably convert ω-3 PUFA compared to AA. Despite this, such dietary manipulation does not result in augmented SPM formation (see *Quantitative Analysis and SPM Levels in Humans*).

Taken together, it becomes obvious that detectable formation of SPMs in leukocytes is mostly restricted to non-physiological conditions such as ionophore-mediated cell stimulation and exogenous supply with fatty acid substrates.

### Lipoxin and Resolvin Formation in Human Macrophages

In contrast to neutrophils, human monocytes/macrophages express AA 15-lipoxygenating paralogues (15-LO1, 15-LO2) depending on their differentiation status. Different 15-LO1 expression levels can be found in M1 and M2 macrophages (Wuest et al., 2012; Ebert et al., 2020). Expression of 15-LO2 is induced after stimulation of M2-differentiated macrophages with the TLR ligands LPS and zymosan as well as hypoxia (Rydberg et al., 2004; Ebert et al., 2020; Von Hegedus et al., 2020). 15-LO1 expression is IL4-dependent and is strongly upregulated during differentiation of monocytes to M2-like macrophages by IL4 (Conrad et al., 1992; Ebert et al., 2020). Since macrophage phenotype switching has been proposed as an important step in the transition of a pro-inflammatory reaction into the resolution phase (Gordon, 2003), the alterations of 15-LO1 and 15-LO2 expression during macrophage phenotype switching have been proposed to be part of a lipid mediator switch from pro-inflammatory lipid mediators such as leukotrienes and prostaglandins to SPMs (Chiang and Serhan, 2017). In addition to 15-LO1 and 15-LO2, macrophages also express 5-LO and FLAP (Ebert et al., 2020). Thus, in principle, these cells should be capable of producing SPMs. However, their SPM forming capacity is very limited. TLR-2 and -4 mediated stimulation of M1 macrophages primarily triggered the release of pro-inflammatory cytokines. Persistent stimulation of human M2 macrophages with LPS induced a coordinated upregulation of 5-LO and 15-LO2 expression (Ebert et al., 2020). 15-HETE but no SPMs were found in the conditioned media of the M2 macrophages and also after fMLF-stimulation of these cells. After stimulation with Ca^2+^ ionophore combined with supplementation of AA, EPA and DHA, M2 macrophages released small amounts of trihydroxy SPMs (RvD1, RvD2, RvE1, LXA_4_, LXB_4_). However, the concentrations of these metabolites were 100–1,000-fold lower than those of LTB_4_ or different monohydroxylated PUFAs. On the other hand, the dihydroxy SPMs RvE2 and RvD5 as well as 5,15-diHETE were formed in stimulated M2 macrophages in detectable amounts (Ebert et al., 2020). Similar results were obtained in another study where the lipid mediator profile in M1 and M2 polarized macrophages in the presence of a pathogenic *E. coli* strain was investigated: Here, consistently detectable RvD5 and MaR1 (7,14-diHDHA) levels were found in stimulated M2 macrophages but the formation of the other SPMs of the D- and E-series of resolvins was low, i.e. >400-fold and 40-fold lower than prostaglandin E_2_ and leukotriene B_4_ (LTB_4_) formation in the corresponding M1 macrophages, respectively (Werz et al., 2018). Similar lipid mediator profiles were reported in two subsequent reports by the same group (Rao et al., 2019; Jordan et al., 2020). An interesting finding of these studies on macrophages was that stimulation of M1 and M2 macrophages leads to a different lipid mediator profile released from the cells which seems to be due to the upregulation of 15-LO1 during M2 polarization and reduction of classical 5-LO metabolites such as 5-HETE and LTB_4_, probably via inhibition of 5-LO activity by the 15-LO reaction products such as 15-HETE (Vanderhoek et al., 1980; Camp and Fincham, 1985). In fact, stimulation of M2 macrophages led to the very prominent release of 15-HETE (Rao et al., 2019; Ebert et al., 2020; Jordan et al., 2020).

From these data, it becomes evident that macrophage polarization to M2 leads to a lipid mediator switch from the 5-LO to the 15-LO pathway. However, leukocytes have a very low capability of synthesizing trihydroxylated SPMs even in the presence of both, 5-LO and 15-LO, so that only 5,15-diHETE, RvD5, RvE2 and MaR1 (a 12/15-LO product) could be consistently detected in macrophages (Figure 1). Whether these levels are biologically relevant remains to be determined. Of note, 5S,15S-diHETE formation by dual lipoxygenase action was already reported 40 years ago (Borgeat et al., 1982), but up to now, this compound has never been classified as an SPM. The corresponding EPA-derived 5S,15S-diHEPE was recently assigned as resolvin E4 (Reinertsen et al., 2021). The relevance of the formed concentrations of either of these compounds to the resolution of inflammation remains to be determined.

The spectrum of the SPMs formed by M2 macrophages is more or less in agreement with the *in vitro* data obtained with purified LO paralogues (see above). Furthermore, it should be kept in mind that even the levels of the more prominent dihydroxylated SPMs formed in leukocytes tend to be significantly lower compared to pro-inflammatory lipid mediators, even if the cells are provided with exogenous substrates and different stimuli.

Based on the currently available experimental data, it appears unlikely that many of the proposed SPMs, especially trihydroxylated SPMs, are formed in leukocytes in sufficient amounts to counteract the functionality of the pro-inflammatory lipid mediators unless AA release is inhibited. However, this would be difficult to achieve, given the numerous cytosolic and secreted phospholipases A2 that can contribute to PUFA release. From the SPMs that involve 5-LO in their biosynthesis, only 5,15-diHETE, RvE2, RvE4 (5,15-diHEPE) and RvD5 seem to be formed in detectable quantities in leukocytes where 5-LO acts as first or second oxygenase. Alternatively, based on studies from platelet-leukocyte interactions, the 5-LO:12/15-LO pathway where LTA_4_ and LTA_5_ are released from leukocytes and further converted to lipoxins via a transcellular mechanism might have the capacity to be a potential source for lipoxins. Studies with 12-LO (Alox12) knockout mice do not support a role of this pathway in the resolution of inflammation (see *SPMs and Lipoxygenase Knockout Data*).

### Autoxidation of PUFAs

Polyunsaturated fatty acids (PUFAs) such as EPA and DHA are susceptible to autoxidation (Frankel, 2005). Compared to enzymatic lipid peroxidation catalyzed by ALOX-isoforms, this process is slow but can be dramatically accelerated many fold in the presence of transition metals or other non-enzymatic catalysts (Leung et al., 2015). When the content of hydroxylated EPA and DHA derivatives was analyzed in a number of commercial fatty acid preparations, monohydroxylated and dihydroxylated derivatives were always detected. 15-HETE, 18-HEPE and 20-HDHA were dominantly found but 5-HETE, 5-HEPE and 4-HDHA were also detected. Since the latter compounds rapidly undergo lactonization, their relative abundance as primary auto-oxidation products is usually underestimated. By contrast, trihydroxy PUFAs such as LXA_4_ and LXB_4_ isomers were never observed in other than trace quantities. However, the presence of mono- and dihydroxy PUFAs in commercial PUFA preparations is a serious problem since these compounds may be used as substrates for trihydroxy-SPM formation. In fact, when commercial AA preparations were incubated with leukocytes or with different recombinant LO isoforms, the formation of lipoxin isomers was sometimes detected. In contrast, with purified AA these products were never observed. Thus, when using exogenous PUFAs to stimulate SPM formation in any cellular and subcellular system, it is highly recommended to purify the fatty acids prior their use.

A feature of non-enzymatic oxidation of PUFAs is the formation of multiple isomeric products (combination of different epimers as well as positional and cis/trans isomers) confounding instrumental analysis if not specifically considered and assessed. It should be noted that SPMs present in a biological sample could arise from autoxidation of either the parent PUFA or from autoxidation of monohydroperoxy PUFAs which were generated nonenzymatically or by the above-mentioned lipoxygenases. In other words, the different oxygenation reactions involved in the (bio)synthesis of double and triple oxygenated SPMs may be catalyzed by enzymes and/or non-enzymatic catalysts. The big difference between enzymatic and non-enzymatic oxygenation reactions is the degree of product specificity. If a given SPM is present as single isomer, its enzymatic origin is most likely. However, if a complex mixture of isomers is found, non-enzymatic oxidation reactions were probably involved.

The complexity of SPM structures with a multitude of isomers is a challenge for the instrumental analysis of these oxylipins (Gladine et al., 2019). For instance, LXA_4_ (5,6,15-triHETE) is often formed together with several isomers (enantiomer, diastereomers, cis/trans isomers and positional isomers). Up to now, only selected isomers have been chemically synthesized and for many of them no authentic standards are currently available. Thus, some of these SPM isomers may overlap during elution in commonly used reversed phase (RP) chromatography. This problem has been addressed for a few SPMs by employing chiral phase chromatography (Ferreiros et al., 2014) or ion mobility spectrometry (Jonasdottir et al., 2015) demonstrating the presence of interfering isomers in biological samples. Unfortunately, these analytical techniques require higher amounts of analytes or have not been implemented in routine analyses. As summarized in *Quantitative Analysis and SPM Levels in Humans* only RP-LC-MS/MS analysis is routinely used for quantification. With respect to SPM isomers formed by autoxidation, one should keep in mind that the chromatographic peaks may contain several isomers, even if the method has been developed and calibrated using a specific isomer available as pure standard.

### Summary on SPM Biosynthesis Pathways

The proposed biosynthetic pathways for most SPMs involve 5-LO (ALOX5) as well as 12/15-LO activities (15-LO1, 15-LO2, 12-LO). Since 5-LO expression is mainly restricted to leukocytes, these cells are supposed to be key players in SPM formation. Biochemical studies with purified lipoxygenases revealed that their capacity for formation of trihydroxylated SPM is extremely low and dihydroxylated SPMs are formed at much lower rates than the classical reaction products (e.g., HETEs, HEPEs, HDHAs and leukotrienes). In accordance with these *in vitro* studies, formation of most SPMs is only detected after stimulation of leukocytes with non-physiological stimuli such as ionophore after addition of exogenous fatty acid substrates. The capacity of leukocytes for the formation of trihydroxylated SPMs is very low. Dihydroxylated SPMs such as RvE2, RvE4 (5,15-diHEPE) and RvD5 can be detected but at levels considerably below those of classical pro-inflammatory oxylipins. Based on the published biochemical and cellular data, the plausibility of at least some of the proposed SPM biosynthetic pathways is questionable. Whether any of them are operable in *in vivo* systems and whether they generate SPMs of functional relevance can only be verified using highly reliable analytical methodology for their detection in complex biological matrices (see *Quantitative Analysis and SPM Levels in Humans*).

## SPMs and Lipoxygenase Knockout Data

The role of lipoxygenases in inflammatory processes has also been investigated by genetic knockout of different mouse Alox paralogues. Knockout of Alox5 has been shown to ameliorate certain inflammatory insults (Chen et al., 1994), protect from pulmonary fibrosis (Peters-Golden et al., 2002), attenuate renal ischemia-reperfusion injury (Patel et al., 2004), lower LPS-induced endotoxemia (Collin et al., 2004), impair the adaptive immune responses during fungal infections (Secatto et al., 2012) and attenuate antibacterial activity and phagocytosis (Bailie et al., 1996). Furthermore, it has been shown that 5-LO plays a pivotal role in lung injury after experimental sepsis (Monteiro et al., 2014). Using FLAP and LTC_4_ synthase knockout mice, it was found that cysteinyl leukotrienes contribute to renal fibrosis and to the progression of chronic kidney disease (Montford et al., 2019). Alox5 knockout mice also showed improved wound healing (Brogliato et al., 2014). Other data suggest that the 5-LO pathway may play a role in the development of neurodegenerative diseases (Joshi and Pratico, 2014). However, in the experimental acute encephalomyelitis (EAE) system, which is considered a suitable mouse model for human multiple sclerosis, Alox5^−/−^ mice developed more severe neurological symptoms than corresponding controls (Emerson and Levine, 2004). The mechanisms behind this observation are still unclear, one possible explanation would be that Alox5 knockout impairs SPM formation which enhances inflammation. However, it should be emphasized that most knockout studies point to a pro-inflammatory role for the 5-LO pathway.

Interestingly, Alox5-knock-in mice which express an AA 15-lipoxygenating 5-LO enzyme mutant (Marbach-Breitruck et al., 2021) are leukotriene deficient, but these animals were not protected from the development of inflammatory symptoms in different mouse inflammation models. Studies on the role of 5-LO in atherosclerosis have delivered controversial results. Initial studies identified 5-LO as a contributor to atherosclerosis development in mice (Mehrabian et al., 2002) which could not be confirmed in later studies [for review, see (Poeckel and Funk, 2010)].

Taken together, it has become evident that the 5-LO pathway is part of the innate and adaptive immune system. It is involved in host defense reactions and promotes inflammatory processes (Peters-Golden et al., 2005; Flamand et al., 2007), but there are leukotriene independent elements in the immune response. This physiological profile is in agreement with many reported functions of the leukotriene receptors BLT1, CysLT1 and CysLT2 (Sasaki and Yokomizo, 2019).

Since 15-LO has been implicated in the lipid mediator switch during human macrophage polarization towards M2, the effects of Alox15 knockout on inflammatory reactions and on the resolution of inflammation are of special interest. In humans, a loss-of-function variant in the ALOX15 gene was shown to protect against the development of nasal polyps and chronic rhinosinusitis (Kristjansson et al., 2019). Furthermore, functional inactivation of the Alox15 gene resulted in reduced expression of pro-inflammatory genes and less severe colitis in the DSS mouse model (Kroschwald et al., 2018), diminished atherosclerosis in ApoE or LDL receptor deficient mice (Cyrus et al., 1999; Rong et al., 2012), reduced airway allergic inflammation in response to ovalbumin (Andersson et al., 2008), improved survival in mouse models of acute lung injury (Rossaint et al., 2012), reduced insulin resistance induced by high fat diet (Sears et al., 2009) and protected hyperlipidemic mice from nonalcoholic fatty acid liver disease (Martinez-Clemente et al., 2010). However, besides these pro-inflammatory effects of 12/15-LO (Alox15), several reports demonstrated an anti-inflammatory role of this enzyme. It was found that 12/15-LO promotes wound healing and host defense as well as counteracts fibroblast activation and fibrosis (Gronert et al., 2005; Kronke et al., 2012; Ogawa et al., 2020). A protective role for 12/15-LO has also been described in arthritis (Kronke et al., 2009) and in IL-33-induced eosinophilic airway inflammation which seems to be at least partially due to the 12/15-LO product 14-HDHA (Miyata et al., 2021). In a context of high EPA and DHA tissue levels, 12/15-LO led to protection from DSS-induced colitis, which was abolished by functional inactivation of the enzyme (Rohwer et al., 2021), further underlining a context-specific pro- or anti-inflammatory role of the enzyme. When human 15-LO1 is overexpressed in transgenic rabbits and in mice, an anti-atherogenic effect was observed (Serhan et al., 2003; Merched et al., 2008). However, the experimental strategy of overexpressing the human 15-LO1 in rabbits and mice deserves more detailed discussion. Like humans, rabbits express an AA 15-lipoxygenating Alox15 orthologue, whereas mice express an AA 12-lipoxygenating enzyme. This means that the transgenic rabbits express their endogenous AA 15-lipoxygenating enzyme and in addition the human transgenic enzyme, which is also AA 15-lipoxygenating. In contrast, the ALOX15 transgenic mice express their endogenous AA 12-lipoxygenating enzyme and in addition the AA 15-lipoxygenating transgenic gene product. These functional differences in the reaction specificity of the endogenous Alox15 orthologues and the transgenic enzymes make the system very complex and hamper straightforward interpretations.

Taken together, it is becoming clear that 12/15-LO can induce both pro- and anti-inflammatory effects which may depend on the expression level of the enzyme(s), on the availability of different substrates and on the regulatory peculiarities of the enzyme in different mammals. The multiplicity and availability of substrates and enzymatic products which are generated and decomposed in a tissue as well as species-specific manner may play an important role for the outcome of these experiments. When it comes to the functionality of Alox15 and Alox15B orthologues, species-specificity is particularly critical since for these enzymes, remarkable functional differences have been described. Moreover, it has never been explored in detail, which role the AA 8-lipoxygenating mouse Alox15B might play in the biosynthetic cascade of SPMs.

Even in those experimental systems in which the formation of SPMs has been reported (Merched et al., 2008; Fredman et al., 2016; Chiang and Serhan, 2017) a causal relation between the formation of these compounds and the observed anti-inflammatory or pro-resolving effects is unclear. There may be statistical correlations, but in most cases, it remains unclear whether the formation of SPMs is the basis for the observed biological effects. Additional knockout of the proposed receptor(s) (see *Proposed SPM Receptors*) would be helpful to dissect the role of individual lipid mediators in the animal models.

The 5-LO:12/15-LO pathway (Figure 1), in which LTA_4_ and LTA_5_ are released from leukocytes and further converted to lipoxins via transcellular mechanisms has been suggested as an effective source for lipoxins (Edenius et al., 1991). Platelet 12-LO has been implicated in the regulation of platelet activation (Tourdot and Holinstat, 2017) and cancer metastasis (Pidgeon et al., 2002). Platelets derived from Alox12 knockout mice showed modified ADP-induced platelet aggregation (Johnson et al., 1998) and the animals experience an increased transepidermal loss of water (Johnson et al., 1999). Moreover, Alox12 knockout inhibited skin carcinogenesis (Virmani et al., 2001) and reduced atherosclerosis and abdominal aneurysm (Allen-Redpath et al., 2019). However, to the best of our knowledge, impairment of inflammatory resolution has never been reported in these mice although the animals have been available for more than 30 years. On the contrary, knockout of Alox12 protected mice from injury-induced neuroinflammation suggesting a pro-inflammatory activity of 12-LO (Li et al., 2019). Thus, at present there is no experimental evidence from Alox12 knockout studies that the 5-LO+12-LO mediated formation of lipoxins may play a significant role in resolution of inflammation *in vivo*.

### Summary on SPM Biosynthesis in Genetically Modified Mice

Since 5-LO has been suggested as key enzyme in the biosynthesis of many SPMs, Alox5^−/−^ mice should not be capable of synthesizing these mediators and should show a defect in the resolution of inflammation. By contrast, most phenotypes of these knockout mice are anti-inflammatory. However, it has to be considered that a possible pro-resolution role of 5-LO might not be observed in the absence of pro-inflammatory 5-LO products in knockout mice. 15-LO can induce both pro- and anti-inflammatory effects which may depend on its expression level and on the availability of different substrates, but it is unclear which 15-LO enzymatic products are responsible for these observations. There is no clear evidence that any of the described lipoxygenase knockouts result in phenotypes which are due to disrupted formation of SPMs.

## Proposed SPM Receptors

### FPR2/ALX

After initial binding studies (Fiore et al., 1992), it was proposed that a close homologue of the formyl peptide receptor 1 (FPR1), FPR2, also named ALX (Brink et al., 2003; Chiang et al., 2006; Ye et al., 2009), is the receptor through which LXA_4_ exerts its effects (Fiore et al., 1994). FPR2 was later shown to serve as a receptor for a large number of ligands belonging to different chemical classes, including both formylated peptides and non-formylated peptides/proteins as well as small molecules (He and Ye, 2017). The initial ligand-receptor pairing of LXA_4_ and FPR2/ALX was based on the demonstration that [^3^H]-LXA_4_ binds to the surface of intact human neutrophils, a binding that was reduced not only by non-labeled LXA_4_ but also by the non-hydrolysable GTP analogue GppNHp (Fiore et al., 1992). [^3^H]-LXA_4_ also specifically bound to isolated neutrophil plasma membranes and to intact lysosomal granules as well as to FPR2/ALX expressing CHO cells that were first freeze thawed to facilitate binding (Fiore et al., 1994). It was also shown that GTPγS affected binding of LXA_4_ to the receptors present on the granule vesicle membrane, and that LXA_4_ increased the GTPase rate in permeabilized CHO cells expressing FPR2/ALX. In this, as well as in subsequent reports, a negative control in which binding and GTPase experiments are performed in mock-transfected cells was missing (Fiore et al., 1994; Chiang et al., 2000). However, it was demonstrated that cells transfected with FPR2/ALX showed a pertussis toxin-sensitive release of arachidonic acid in response to LXA_4_, while mock-transfected CHO cells did not (Fiore et al., 1994), even if in these experiments, concentration-dependency and EC_50_ values of the LXA_4_ effect were not determined.

Several other reports demonstrated that cells responded to LXA_4_ in a FPR2/ALX-dependent manner. For instance, in RBL-2H3 cells as well as in human monocytes, LXA_4_ induced a transient increase in [Ca^2+^]_i_ depending on FPR2/ALX (Maddox et al., 1997; Bae et al., 2003). The rise in [Ca^2+^]_i_ was rather weak as compared to the FPR2/ALX-selective peptide agonist WKYMVM (Bae et al., 2003), and LXA_4_ did not induce ERK phosphorylation whereas WKYMVM did so (Bae et al., 2003). In addition, siRNA-mediated knock-down of FPR2/ALX blocked LXA_4_-induced Ca^2+^ transients in rat goblet cells (Hodges et al., 2017). Barnig et al. showed that LXA_4_ can act on NK cells to induce eosinophil apoptosis as well as on type 2 innate lymphoid cells (ILCs) to decrease IL-13 release, which in both cases was inhibited by the FPR2 receptor antagonist WRW4 (Barnig et al., 2013). While these data suggest that LXA_4_ acts as an FPR2/ALX agonist, several other reports were not able to confirm a role of FPR2/ALX as an LXA_4_ receptor. Two groups reported independently that LXA_4_ was unable to induce effects in HL60 cells transfected with FPR2/ALX or in human neutrophils known to express FPR2/ALX endogenously, whereas WKYMVM was active (Christophe et al., 2002; Planaguma et al., 2013). Subsequent studies showed that LXA_4_ from at least two different commercial sources was not able to induce FPR2/ALX-dependent effects in neutrophils (Forsman and Dahlgren, 2009; Forsman et al., 2011). Failure of LXA_4_ to induce an increase in [Ca^2+^]_i_ in the presence of FPR2/ALX was also reported by other groups (Hanson et al., 2013; Bang et al., 2018).

A large number of experiments using receptor antagonists have been published in order to test a direct link between effects of SPMs and FPR2/ALX. However, in many of these studies antagonistic peptides that preferentially inhibit FPR1 rather than FPR2, such as Boc-1, Boc-MLF, or Boc-2, BocFLFLF have been used (Krishnamoorthy et al., 2010; Wu et al., 2014; Zhang et al., 2017; Mai et al., 2018; Yang et al., 2018; Lyngstadaas et al., 2021).

Regarding alternative receptor down-stream signaling events, a publication reported that LXA_4_ increased the luminescence signal using a complementation β-arrestin assay in FPR2/ALX-expressing cells (Krishnamoorthy et al., 2010). In the same study, the authors also described RvD1 as an agonistic ligand of FPR2/ALX, and knock-down of FPR2/ALX led to strongly reduced effects of RvD1 on macrophages (Krishnamoorthy et al., 2010; Lee and Surh, 2013). While these data suggest that LXA_4_ may act as a biased FPR2/ALX agonist, several other reports were not able to confirm such a role of FPR2/ALX as an LXA_4_ receptor. Two subsequent studies showed that recruitment of β-arrestin was not induced by LXA_4_ from at least two different commercial sources whereas peptide agonists specific for FPR2/ALX induced translocation of β-arrestin (Forsman and Dahlgren, 2009; Forsman et al., 2011). Similarly, in HEK293 and CHO cells expressing FPR2/ALX, no effect of LXA_4_ from different sources could be observed on β-arrestin 2 membrane translocation, whereas again the FPR2/ALX agonist WKYMVM was active (Hanson et al., 2013). This study also ruled out that LXA_4_ was degraded before or during the experiment by performing mass spectrometry determination of LXA_4_ concentrations in the assay buffer after completion of the experiment.

More recently, it was shown that so called aspirin-triggered 15-epi-lipoxin A_4_ (ATL), an isomer of LXA_4_ with unclear *in vivo* relevance, interacted in a complex manner with FPR2/ALX, using a modified version of FPR2/ALX, which allows determination of conformational changes of FPR2/ALX by fusing FPR2/ALX with CFP and by inserting a CCPGCC motif into the first or third intracellular loop (Ge et al., 2020). ATL, which has not yet been shown to be formed under *in vivo* conditions, induced only a small increase in [Ca^2+^]_i_ and of β-arrestin 2 membrane translocation at concentrations higher than 1 µM. However, it had a partial agonistic effect on the inhibition of cAMP accumulation already at a concentration of 1 nM. In addition, the authors found opposing effects on the FPR2/ALX conformation at concentrations between 1 and 100 pM as well as 100 pM and 1 µM. At the same time LXA_4_ was unable to displace WKYMVM from the receptor at concentrations up to 1 μM, whereas ATL was able to affect binding of WKYMVM and WKYMVM downstream signaling at much lower concentrations. The authors concluded that ATL at subnanomolar concentrations functions as an inverse agonist, most likely in an allosteric fashion, whereas it functions as an orthosteric partial agonist at much higher concentrations.

In terms of biology of the receptor, various ligands of different chemical natures can have similar or opposite cellular effects. For example, while LXA_4_, RvD1 and Cmp43 have anti-inflammatory properties, formyl peptides produce a pro-inflammatory response. Since the known signaling events that take place upon receptor activation such as [Ca^2+^]_i_ mobilization are shared for example by Cmp43 and formylated peptides, simple signaling differences have not yet been identified that are responsible for the functional differences at the cellular level. To explain this inconsistency, Cooray et al. have proposed that each ligand could stabilize distinct conformations of FPR2/ALX with different outcomes in terms of dimerization of the receptor and subsequent activation of signaling pathways (Cooray et al., 2013). However, there is no experimental evidence in support of this conjecture.

Several studies explored the potential role of FPR2/ALX as a mediator of LXA_4_ effects *in vivo* using Fpr2/Alx-deficient mice. The establishment of a relevant mouse model is, however, problematic since initial characterizations identified both FPR2/ALX and FPR3 as the mouse receptor for LXA_4_ (Takano et al., 1997; Vaughn et al., 2002). The first study using knock-out mice later turned out to have employed an (Fpr2/Alx)/Fpr3 double knock-out model (Dufton et al., 2010). In addition, subsequent studies showed that mice lacking FPR2/ALX have already a phenotype under basal conditions, which may be due to loss of basal (FPR2/ALX)/FPR3 activity or loss of the effects of other (FPR2/ALX)/FPR3 receptor agonists such as Cmp43 and the endogenous ligands annexin I and several non-amyloidogenic peptides (He and Ye, 2017). In another study it was shown that deletion of FPR2/ALX has an anti-inflammatory effect in an airway inflammation model (Chen et al., 2010). This indicates that even if endogenous LXA_4_ can activate this receptor and exert an anti-inflammatory effect, it cannot overcome the pro-inflammatory effects of other endogenous FPR2/ALX ligands, suggesting that the interaction of LXA_4_ with FPR2/ALX is of limited *in vivo* relevance, at least in the context of airway inflammation. It should also be noted that, in addition to the prominent differences that exist in gene expansion of formyl peptide receptors (FPRs) between humans and mice, there is also increasing evidence indicating that human FPRs and their murine counterparts differ substantially with respect to their ligand-binding profiles, as illustrated by the fact that some of the potent and selective antagonists for the FPRs lack effects on the mouse receptors, and an earlier described FPR2/ALX inhibitor/antagonist actually activates FPR2/ALX (Winther et al., 2018). No such comparative studies of mice and men involving SPMs have been performed, which highlights the importance in future research studies of choosing appropriate ligands when designing animal experiments. Together, this makes the interpretation of differential effects of LXA_4_ in animal knock-out models difficult (Dufton et al., 2010; Maderna et al., 2010; Norling et al., 2012; Brancaleone et al., 2013; Vital et al., 2016; Petri et al., 2017). There are, thus, still conflicting data regarding the specific role and signaling properties of FPR2/ALX in the action of LXA_4_ and the related lipid ATL, and this also goes for the action of RvD1 (see the *GPR32* dealing with GPR32 for more details).

### GPR18

A β-arrestin recruitment assay was used to screen a large number of RvD2 receptor candidates, leading to the nomination of GPR18 (Chiang et al., 2015). Specific binding of [^3^H]-RvD2 to CHO cells expressing human GPR18 with a K_D_ value in the lower nanomolar range was shown, but negative controls using non-transfected cells were not reported. However, knock-down of GPR18 in macrophages resulted in loss of RvD2-induced cAMP formation and other cellular effects (Chiang et al., 2015), although previous data from the same group suggested that RvD2 acts through a G_i_-coupled receptor based on pertussis toxin sensitivity of RvD2 effects on endothelial cells (Spite et al., 2009). Also, the ability of RvD2 to exert anti-inflammatory and protective activity *in vivo* was lost or strongly reduced in GPR18-deficient mice (Chiang et al., 2015). The same group later provided additional evidence for an *in vivo* role of GPR18 as a receptor for RvD2 in various models of infectious inflammation (Chiang et al., 2017). So far, the activity of RvD2 as an agonist of GPR18 has not been reported by an independent laboratory, but one independent study was unable to reproduce agonism for RvD2 in a β-arrestin recruitment assay (Schoeder et al., 2020).

Previously, GPR18 has also been described as a receptor for *N*-arachidonoyl glycine (NAGly) as well as for Δ^9^-tetrahydrocannabinol (Kohno et al., 2006; Mchugh et al., 2010; Mchugh et al., 2012). However, agonism of these two ligands for GPR18 was not reproduced in three reports, which used β-arrestin assays (Yin et al., 2009; Southern et al., 2013; Schoeder et al., 2020).

### GPR32

RvD1 has been shown to interact with neutrophils and to have anti-inflammatory and pro-resolving effects fairly similar to those of LXA_4_. Based on a screen for candidate GPCRs able to mediate inhibition of TNFα-stimulated NFκB activity by RvD1, not only FPR2/ALX but also GPR32 was found to function as a receptor for RvD1 (Krishnamoorthy et al., 2010). GPR32 was shown to have an amino acid sequence partly shared with the FPRs (i.e., 39 and 35% amino acid identity with FPR1 and FPR2/ALX, respectively). Screening data were confirmed by showing that RvD1 increased β-arrestin recruitment to the plasma membrane in cells expressing either GPR32 or FPR2/ALX. In the same study, the authors also showed that RvD1 increased phagocytosis by macrophages, and that this effect was not obtained after knock-down of GPR32 (Krishnamoorthy et al., 2010; Krishnamoorthy et al., 2012). The same group later reported that RvD3 and RvD5 are also able to increase β-arrestin activity in CHO cells overexpressing human GPR32. However, control experiments using untransfected cells were not reported (Chiang et al., 2012; Dalli et al., 2013). In addition, both reports demonstrated that RvD3 and RvD5 show increased activity to stimulate macrophage phagocytosis after transfection of cells with GPR32, but no knock-down control data were reported (Chiang et al., 2012; Dalli et al., 2013). GPR32 has also been found to recruit β-arrestin in response to LXA_4_ and the dual FPR1/(FPR2/ALX) agonist Cmp43, and these ligands were as potent as RvD1 (Krishnamoorthy et al., 2010). Studies in mice lacking GPR32 have not been possible since GPR32 is a pseudo-gene in mice and rats (Haitina et al., 2009). An ability of RvD1, other D-series resolvins, LXA_4_ or Cmp43 to activate GPR32 has not been reported by an independent laboratory so far, and agonism of RvD1 for GPR32 could not be replicated in a study using a different assay system (Foster et al., 2019), or in studies using the same β-arrestin recruitment assay (Southern et al., 2013).

### Chemerin Receptor 1

In a small candidate screen for GPCRs mediating inhibition of NFκB-dependent luciferase activity by RvE1, ChemR23 was reported to function as a receptor for RvE1 (Arita et al., 2005). In two publications by the same group, it was demonstrated that RvE1 bound to CHO cells transfected with ChemR23 but not to untransfected cells, and that RvE1-induced inhibition of IL-12 production by dendritic cells was significantly reduced after knock-down of ChemR23 (Arita et al., 2005; Arita et al., 2007). Specific binding of RvE1 to ChemR23 and a role of ChemR23 in typical GPCR downstream signaling events in response to RvE1 has not yet been reported by an independent group. It has been shown by an independent publication that RvE1 inhibited oxLDL uptake by peritoneal macrophages and that this effect was lost in peritoneal macrophages from ChemR23-deficient mice (Laguna-Fernandez et al., 2018).

### BLT1

In addition to ChemR23, RvE1 has also been reported to be a partial agonist of the leukotriene B_4_ (LTB_4_) receptor (BLT1) having relatively low potency as compared to LTB_4_ (Arita et al., 2007). RvE1 showed specific binding to human neutrophils and HEK cells overexpressing BLT1 and decreased forskolin-induced cAMP formation in a BLT1-dependent manner. The same group reported later that also RvE2 binds to HEK cells overexpressing BLT1 and to reduce LTB_4_-induced β-arrestin signaling (Oh et al., 2012). These findings suggest that RvE1 and RvE2 induce their effects through a partial agonism at the BLT1 receptor thereby dampening the pro-inflammatory effects of LTB_4_. Evidence for an interaction between BLT1 and RvE1 has not yet been reported by other independent groups.

### Other Receptors

Several other receptors for SPMs have been proposed by single publications. LXA_4_ was reported to function as a positive allosteric modulator of cannabinoid receptor 1 (CB1) (Pamplona et al., 2012). This was based on the observation that LXA_4_ only partially displaced a specific ligand from the CB1 receptor while enhancing the affinity of anandamide and other synthetic CB1 agonists for CB1. Consistent with this, LXA_4_ potentiated effects of CB1 agonists without showing any effect on its own. In addition to several GPCRs, LXA_4_ has also been proposed to interact with nuclear receptors. One report suggests that LXA_4_ functions as an agonist of the aryl-hydrocarbon receptor (AhR) (Schaldach et al., 1999). In a competition binding assay, LXA_4_ displaced a radio-labeled AhR ligand. However, direct binding of LXA_4_ to AhR was not demonstrated. Another publication reported that LXA_4_ activates the estrogen receptor (ER) (Russell et al., 2011). The report showed that LXA_4_ displaced estrogen from its receptor and induced an increase in ER transcriptional activity independent of AhR or FPR2/ALX. However, no negative control experiments were performed.

Neuroprotectin D1 (NPD1) was reported to interact with the orphan receptor GPR37 (Bang et al., 2018). NPD1 was shown to induce calcium mobilization in a GPR37-dependent manner with an EC_50_ in the low nanomolar range. Binding of NPD1 to GPR37 was analyzed using a dot blot assay. Since the ligand is not labeled and solubilized cellular proteins were tested, caution is warranted when interpreting the data. However, NPD1 was shown to increase the phagocytosis in macrophages in a GPR37-dependent manner.

After conducting a screen of orphan GPCRs using the β-arrestin recruitment assay, maresin 1 (MaR1) was reported to interact specifically with the G-protein-coupled receptor LGR6 (Chiang et al., 2019). MaR1 induced an increase in cAMP and β-arrestin recruitment in LGR6 overexpressing cells but not in mock transfected cells. A radiolabeled methylester of MaR1 was shown to bind to CHO cells expressing LGR6. Binding of the radiolabeled ligand in mock transfected cells was not reported. However, MaR1-induced phagocytosis in human macrophages depended on LGR6.

After conducting another screen of orphan GPCRs using the β-arrestin recruitment assay, ω-3 docosapentaenoic acid-derived resolvin D5 (RvD5n-3 DPA) was reported to be able to function through various GPCRs, of which GPR101 mediated the strongest effect (Flak et al., 2020). Radiolabeled RvD5n-3 DPA bound to HEK cells overexpressing Gpr101. However, a negative control by testing the binding of labeled RvD5n-3 DPA to untransfected cells was not shown. Several effects of RvD5n-3 DPA on macrophages were affected by knock-down of GPR101.

### Summary on SPM Receptors

Generally, it has been difficult to describe the interaction of lipids with putative receptors due to their physicochemical properties, including nonspecific binding to proteins, formation of micelles, detergent or surfactant effects as well as indirect effects due to unspecific interactions with membranes. Particular caution has therefore to be taken before it can be concluded that a certain lipid ligand receptor pair exists. A good starting point for a rigorous analysis of such interactions are the published recommendations for new pairings of ligands and GPCRs (Davenport et al., 2013; Laschet et al., 2018). These include that two or more refereed papers from independent research groups should demonstrate activity of the ligand at the receptor with a potency consistent with a physiological function. The experiments need to be properly controlled, e.g., exclusion of unspecific effects in the absence of the receptor. In addition, it is highly recommended that both radio-ligand binding and functional assays should be employed, both *in vitro* and in native tissues/cells. If available, well validated selective agonists should mimic and selective antagonist as well as allosteric modulators should block/modulate the action of the endogenous ligand. Naturally, the putative endogenous ligand should be present in tissues in appropriate concentrations, and a plausible mechanism for the proposed ligand to reach physiologically significant concentration in tissues expressing its cognate receptor should be provided. Finally, deleting the gene encoding the receptor in mice, use of naturally occurring deletion mutations in human tissues or RNA silencing or other techniques to knock down receptor expression should be used to provide further evidence for the proposed ligand-receptor pairing. By these criteria, endogenous receptors for SPMs remain to be validated.

## Quantitative Analysis and SPM Levels in Humans

There are two major requirements for the assignment of the biological function of a given lipid mediator in inflammatory resolution which partly depend on each other: i) The cells involved in this process must exhibit a sufficiently high biosynthetic capacity for the formation of these SPMs. ii) These lipid mediators must reach local concentrations which are high enough to activate their receptor(s) and to counteract the bioactivity of the pro-inflammatory mediators that are formed in parallel. The quantitative analysis of SPMs is indispensable for assessing both requirements.

**FIGURE 2:**
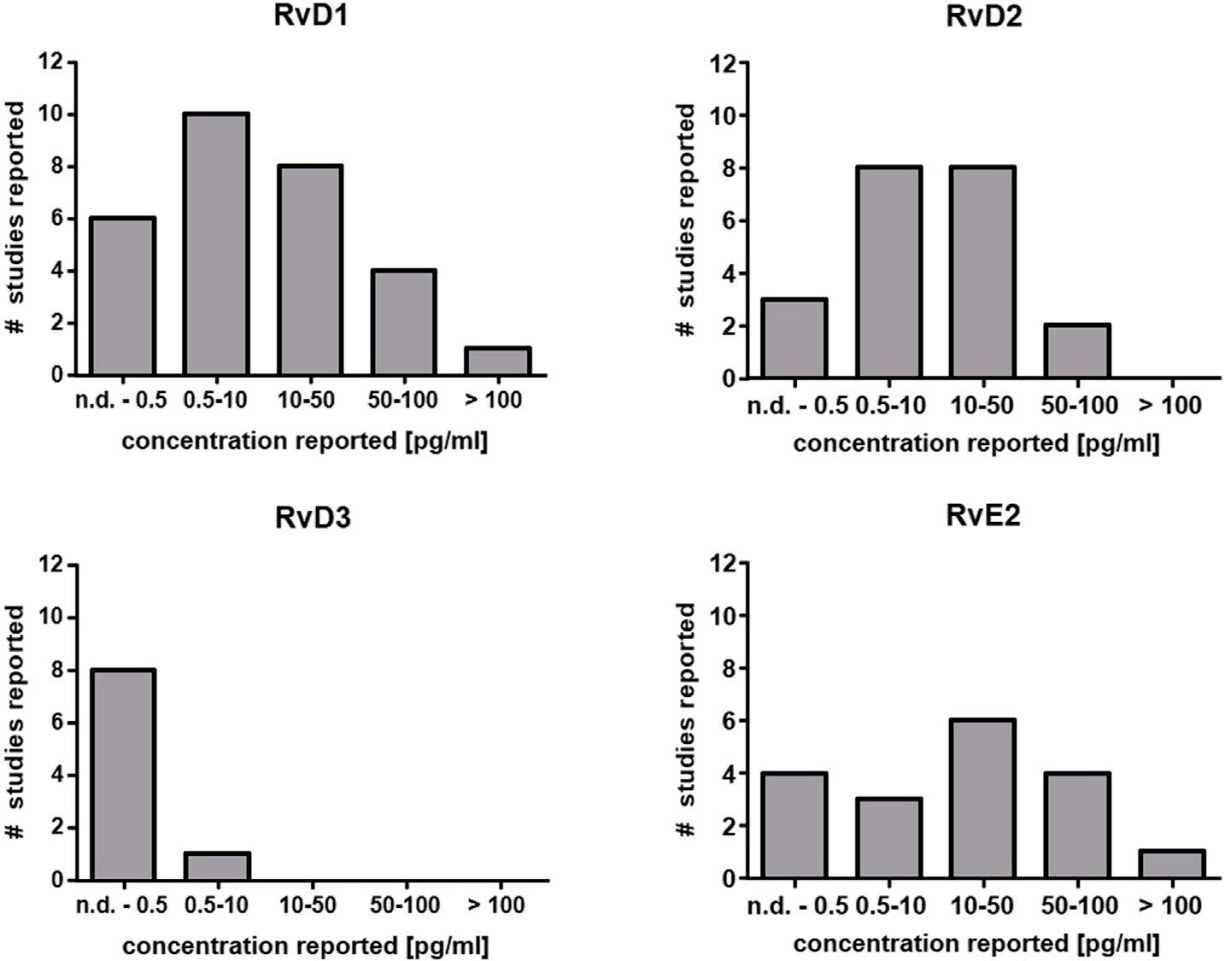
Reported concentration ranges of selected SPMs in human plasma. Based on the comprehensive review from Calder (Calder, 2020) the number of studies and the detected concentration range are summarized for RvD1, RvD2, RvD3 and RvE2 in plasma from human subjects. In the majority of the studies the concentration is low (<50 pg/ml) which is close to the detection limit of several methods or below (Table 2).

The capacity for SPM generation under optimal circumstances in leukocytes even after activation with powerful stimuli is low (*In Vitro Formation of Lipoxins and Resolvins*). Consistently, very low levels have been reported or SPMs cannot be detected at all in human plasma (Figure 2) (Calder, 2020). Since eicosanoids and other oxylipins are believed to predominantly act as autocrine or paracrine mediators, their local concentration in tissues is of greater biological relevance than that in plasma. Unlike prostanoids which are found in tissues such as colon at high levels (ng/g) (Gottschall et al., 2018; Rohwer et al., 2020), the reported tissue concentrations of SPMs are also low, i.e. in the range of pg/g (Serhan, 2017a). Thus, very sensitive analytical methods are required for the reliable quantification of SPMs in biological samples.

### Enzyme-Linked Immunosorbent Assays

Several enzyme-linked immunosorbent assays (ELISAs) have been developed for different types of SPMs. They are commercially available and are frequently used. However, taking into account the large number of structurally similar SPMs and other oxylipins (Willenberg et al., 2015), it is unlikely that these kits reach the required selectivity. They have been tested for selected SPM isomers but it remains unclear to which extent they pick-up alternative isomers. For instance, an ELISA developed for 15*-epi*-LXA_4_ cross reacts with LXA_4_ (Fedirko et al., 2017). However, it remains unclear to which extent geometric double bond isomers are also quantified. Moreover, various oxylipins such as 15-HETE, 5,15-diHETE and LXA_4_ on the one hand and 5-HETE, 5,15-diHETE and LXB_4_ on the other share at least single epitopes. Since the epitopes used for the different antibodies have not been carefully characterized the degree of cross-reactivity with other SPMs, never mind other lipids, cannot be predicted. Thus, detailed cross-reactivity studies are required for reliable analytical data. For the interpretation of ELISA-based SPM data another point must be considered: It has been reported that 5,6-diHETE cross reacts by 5% in the Neogen LXA_4_ kit. Since LXA_4_ is found in biological samples at much lower abundance than 5,6-diHETE, the documented LXA_4_ levels may at least in part be due to 5,6-diHETE. Another example is RvD1, its plasma concentration was ∼30 pg/ml determined by LC-MS. In contrast, by using ELISA assays, levels above 2000 pg/ml were found, as summarized recently (Calder, 2020). In summary, ELISAs for SPMs are unreliable for complex biological matrices.

### LC-MS Analysis of SPMs

The required sensitivity and selectivity for oxylipin analysis including SPMs can only be reached using chromatography coupled to mass spectrometry (MS) (Willenberg et al., 2015; Gladine et al., 2019). To date, only liquid chromatography coupled *via* electrospray ionization (ESI) to tandem MS is frequently used for quantitative SPM analysis. In fact, the techniques employed are almost identical using commercially available “ultra high” performance (sub-2 µm particle filled) chromatographic RP-columns with a diameter of 2.1 mm and flow rates of about ∼300 μl/min (Table 2; Supplementary Table S1), “standard” ion sources and triple quadrupoles as analyzers operating in selected/multiple reaction monitoring mode (MRM, Table 2) (Willenberg et al., 2015; Gladine et al., 2019). Thus, with nearly identical instrumentation and method parameters one would assume that the sensitivity for the detection is comparable between different labs.

**TABLE 2:** Sensitivity of state-of-the-art analysis of SPMs using LC-MS/MS.

Laboratory	References	Instrument LLOQ		LLOQ plasma/serum/fluid	Instrument	LLOQ definition
		pg on column	pg/ml vial	pg/ml		
Dalli	Colas et al. (2014)	0.05–0.22^a^ ^,^ ^c^, d_5_-LXA_4_ 0.05, d_5_-RvD2, 0.09	1.3–5.5, LXA_4_ 1.3, RvD2 2.3	0.05–0.22, d_5_-LXA_4_ 0.05, d_5_-RvD2, 0.09	Sciex 6500	
Dalli	Gomez et al. (2020), Koenis et al. (2021), 10.21203/rs.3.pex-1147/v1	0.05–5.00, LXA_4_ 0.1, RvD2 0.1^b^ ^,^ ^c^	1.4–142, LXA_4_ 2.9, RvD2 2.9	0.05–5.00, LXA_4_ 0.1, RvD2 0.1	Sciex 5500	
Dennis	Dumlao et al. (2011), Deems et al. (2007), Quehenberger et al. (2011)	1^a^, LXA_4_ 1	25	2.8	Sciex 4000	S/N ≥ 3 (n = 3) (LOD)
Geisslinger	Toewe et al. (2018)	1–2, LXA_4_ 2, RvD2 2	100–200, LXA_4_ 200, RvD2 200	25–50, LXA_4_ 50, RvD2 50	Sciex 5500	S/N ≥ 10, ± 20% accuracy and precision
Giera	Jónasdóttir et al. (2015)	0.5, LXA_4_ 0.5, RvD2 0.5	25, LXA_4_ 25, RvD2 25	200	Sciex 6500	S/N > 10
Giera	Jonasdottir et al. (2017), Giera et al. (2012)	0.4, LXA_4_ 0.4	10^d^, LXA_4_ 10, RvD2 no calibration	Synovial fluid 12	Sciex 6500	S/N > 10
Hammock	Yang et al. (2009)	0.21	21	8	Sciex 4000	S/N ≥ 10
Hersberger	Hartling et al. (2021)	0.002–0.063, LXA_4_ 0.008, RvD2 0.002	0.2–6.3, LXA_4_ 0.8, RvD2 0.2	0.4–12.5, d_5_-LXA_4_ 0.4, d_5_-RvD2 3.2^c^	Sciex 6500+	S/N > 10
Mori	Mas et al. (2012)	6	250	25	Thermo TSQ Quantum	S/N ≥ 10
Newmann	Pedersen et al. (2021)	0.2–1, LXA_4_ 0.4, RvD2 0.8	40–199, LXA_4_ 80, RvD2 159	201–995, LXA_4_ 398, RvD2 794	Sciex 6500	3 × *t*_n-1,0.95_ × STD^e^
Nicholson	Wolfer et al. (2015)	0.05–5, LXA_4_ 1.3, RvD2 0.5	10–1000, LXA_4_ 260, RvD2 100	12–1200, LXA_4_ 312, RvD2 120	Waters Xeno TQS	S/N > 5, intraday RSD <20% (n = 4), accuracy ±20%
Ramsden	Yuan et al. (2018)	1–5, LXA_4_ 2, RvD2 5	100–500, LXA_4_ 200, RvD2 500	20–100, LXA_4_ 40 RvD2 100	Sciex5500	S/N > 5, intraday RSD <20% (n = 4), accuracy ±30%
Schebb	Kutzner et al. (2019)	0.6–3.6, LXA_4_ 0.6, RvD2 1.4	61–360, LXA_4_ 61, RvD2 141	6–36, LXA_4_ 6, RvD2 14	Sciex 6500	S/N ≥ 5, ±20% accuracy
Werz	Werner et al. (2019), Werner et al. (2020)	0.195–1.56, LXA_4_ 0.195, RvD2 1.56^a^	19.5–156, LXA_4_ 19.5, RvD2 156^a^	1–8, LXA_4_ 1, RvD2 8^a^	Sciex 5500	S/N > 3, >5 data points (LOD)
Zhu	Wang et al. (2020)	0.18–4.5, LXA_4_ 0.18, RvD2 0.9	1.8–45, LXA_4_ 1.8, RvD2 9	5.4–135, LXA_4_ 5.4, RvD2 27	Agilent 6470	S/N > 7

The reported sensitivity for all covered SPMs and exemplary LXA_4_ and RvD2 is summarized. Shown is the lower limit of quantification of the instrument (LLOQ on column and the corresponding concentration), the effective LLOQ for liquid biological samples in pg/ml, as well the instrument and method used for definition of the LLOQ during method validation. Please note, that this is a simplified table. An extended version of the table including all required information and more method parameters is provided in the supplemental information (Supplementary Table S1).

a Only LLOD is given in the publication.

b Not specified between LLOD/LLOQ.

c Determined in plasma matrix.

d Lowest calibration level injected.

e LLOD/LLOQ was determined based on a significant change (one-tailed *t*-test) in the sensitivity between successive calibration standards using the standard deviation (STD) of the concentration level significantly different than the preceding concentration level and the t-distribution (t-value: one-tailed, 95% confidence).

However, as shown in Table 2, the lower limit of quantification (LLOQ) - which is the lowest concentration that can be reliably quantified in a sample - varies between 0.05 and 6 pg on column. In case of LXA_4_ and RvD2 this means, with an LLOQ of 0.1 - 2 or 0.1–6 pg on column, that the very same instrumentation and equipment appears to be 20–60 fold more sensitive in one lab compared to another.

In our hands, the state-of-the-art instruments operated as described above reach a sensitivity of 0.5–5 pg on column for SPMs, in case of LXA_4_ 0.6 pg and RvD2 1.4 pg on column (Kutzner et al., 2019). In fact, this is the same range as for other oxylipins (Gladine et al., 2019) which is consistent with their structural similarity.

If methods are dramatically more sensitive (which means at least factor 5, not 2-3 which could easily depend on the skills of the person who optimized separation and detection, or on the performance of the specific instrument) there must be another methodologic explanation. This could reflect for example the use of nano-LC, i.e., chromatographic separation at low flow rates enhancing ionization efficacy (Wilson et al., 2015). However, probably due to the lack of robustness this has not been frequently applied in SPM analysis. Recently, Hartling et al. described a method with an LOQ of 8 fg for LXA_4_ and 2 fg for RvD2 on column. Unlike other methods they used a base modified LC solvent, thus enhancing the ionization process in negative ESI mode. This alternative analytical technique is most probably the reason why their method is 100-fold more sensitive (Hartling et al., 2021).

However, Colas et al. (2014) and Koenis et al. (2021) report a sensitivity which is 5–10 fold better (0.05–5 pg on column, 0.1 pg for LXA_4_ and RvD1) than all the other methods (Table 2) using almost the same conditions. This can only be explained by a different definition of the sensitivity, i.e., the LLOQ. Commonly for LC-MS, the LLOQ (*LLOQ-definition I*) is defined by the height of the peak compared to the noise as well as the accuracy of the calibration at this concentration (European medicines Agency, 2011; US Food and Drug Administration, 2018). This assures that not only a signal of the compound is detected beyond doubt (which would be the limit of detection), but also allows calculation of a meaningful concentration with acceptable variation and precision. However, in the field of SPMs several groups use a different definition of the LLOQ (*LLOQ-definition II*), in which the ratio between peak and noise is irrelevant (Colas et al., 2020; Koenis et al., 2021): If the peak exceeds i) a threshold intensity (2,000 counts), ii) a minimum of 4 data points [12–20 points are commonly used for peak definition in LC-MS/MS (European medicines Agency, 2011; US Food and Drug Administration, 2018)] and iii) if the MS/MS spectrum contains ions matching the *m/z* of the SPM fragments, then the peak is integrated and used for quantification (Colas et al., 2020). Using this technique, peaks are considered which would be regarded as too low to determine a reliable concentration by common *LLOQ-definition I*. Particularly in extracts from biological samples, noise can easily exceed the threshold set (e.g., 2,000 cps over four data points) because of the sensitivity of the instruments. The fragment spectra of these background ions contain a variety of nonspecific ions, some of which may have similar *m/z* values as those derived from SPMs (Kutzner et al., 2020). This is illustrated by the chromatograms shown by Gomez et al., e.g., for RvD2, RvD5, RvD6 and LXA_4_, where the integrated peaks are hardly distinguishable from noise (Gomez et al., 2020) and the method used here, which is widely applied to SPM analysis, has been criticized (O'Donnell et al., 2021). Thinking about the quantification of pharmaceuticals (which are not so different from SPMs with respect to molecular weight, functional groups and polarity), one should be aware that the *LLOQ-definition II* is unacceptable for method validation by either the US Federal Drug Administration or the European Medicines Agency (European medicines Agency, 2011; US Food and Drug Administration, 2018).

Regarding the power to detect SPMs in blood and tissue, the sensitivity of the method also depends on an appropriate sample preparation. This is critical both to reduce interfering matrix compounds such as phospholipids (Ostermann et al., 2015), and for pre-concentration. Commonly applied solid phase extraction (SPE) allows reconstitution in a lower volume compared to the initial sample volume facilitating effective LLOQs which are 10- to 25-fold better than for the injected solution (Table 2). If this step is omitted and samples are injected following protein precipitation, low LLOQs are sacrificed making the detection of SPMs generally impossible (Jonasdottir et al., 2017; Pedersen et al., 2021). However, online-SPE methods may allow injecting enough sample volume to gain sensitivity (Wang et al., 2020). Of course, the volume injected, i.e., the amount/percentage of the extracted biological material/volume of plasma (Table 2), impacts the effective sensitivity of SPM detection in biological materials. Pre-concentration is again limited by the available sample volume and even more so by interfering co-extracted matrix components disturbing the detection of SPMs (Ostermann et al., 2015). However, data on extraction efficacy and ion suppression as well as method details and validation data are missing in most reports describing the analysis of SPMs, making the evaluation of the adequacy of the methods difficult.

Consistent with the sensitivity of the analysis (Table 2; Supplementary Table S1) and the described levels (Figure 2), several groups failed to detect SPMs in plasma of healthy individuals (Jónasdóttir et al., 2015; Skarke et al., 2015; Kutzner et al., 2019). Even under conditions anticipated to maximize their formation such as supplementation with their putative ω-3 PUFA precursors by fish oil, SPMs could not be detected (Skarke et al., 2015; Ostermann et al., 2019). Thus, the supplementation of increasing doses of the fish oil to healthy volunteers lead to a dose dependent shift from ω-6 to ω-3 oxylipins, while SPMs were largely undetectable (Skarke et al., 2015; Ostermann et al., 2019). Even after administration of an acute inflammatory stimulus, i.e. bacterial lipopolysaccharide, to such volunteers, SPMs were unaltered in the inflammatory or resolution phase (Skarke et al., 2015). Similarly, Mazaleuskaya et al. largely failed to detect SPMs in stimulated whole blood from healthy volunteers (Mazaleuskaya et al., 2016). Elevated SPM concentrations have been detected in samples from patients suffering from severe inflammation such as COVID-19 or septic shock. However, this also coincided with a strong increase in all oxylipins including pro-inflammatory eicosanoids (Kutzner et al., 2019; Archambault et al., 2021).

### Summary on Quantitative Analysis and SPM Levels

The capacity of tissues to form SPMs is very low and tissue or cellular capacity to generate traditional eicosanoids and other oxylipins greatly exceeds actual rates of SPM biosynthesis. As such it is not surprising that SPM levels in biological fluids would be at or below the limits of detection of the most sensitive MS instrumentation. The specificity of the most commonly applied approach on SPM detection (Dalli and Serhan, 2012; Colas et al., 2014; Dalli et al., 2017; Gomez et al., 2020; Koenis et al., 2021) is not in accordance with internationally agreed standards and has recently been fundamentally questioned (O'Donnell et al., 2021). It remains to be established that SPMs are formed *in vivo* at concentrations of relevance to the resolution of inflammation.

## Conclusion

The resolution of inflammation is now considered as an active process, which coincides with a switch in the profile of cytokines, lipid mediators and other signaling molecules. Some prostaglandins and leukotrienes are typically pro-inflammatory lipid mediators, others have an anti-inflammatory effect. SPMs such as lipoxins and resolvins which are biosynthesized via the 5-LO plus 12/15-LO pathway, have been suggested to play important roles in the resolution of inflammation. However, *in vitro* studies with purified enzymes and studies with leukocytes as a source of 5-LO revealed that many of the lipoxins and resolvins of the D- and E-series are only formed in minute amounts, if at all, since their biosynthetic precursors are hardly accepted by human 5-LO as a substrate for SPM formation, calling into question whether the proposed schemes on their enzymatic formation are biologically plausible. Deletion of their putative biosynthetic enzymes have failed to provide evidence consistent with a role for SPMs in the resolution of inflammation. Evidence that resolvins and lipoxins exert their effects through specific receptors remains controversial and incomplete. Finally, evidence that SPMs are formed in biologically active concentrations in humans that promote the resolution of inflammation remains to be provided.
