# Supplementary material for: Formation, Signaling and Occurrence of Specialized Pro-Resolving Lipid Mediators—What is the Evidence so far?
Source: Front Pharmacol. 2022 Mar 2;13:838782. doi: 10.3389/fphar.2022.838782 (PMC8924552; doi:10.3389/fphar.2022.838782)
Supplement: Supplementary file 1 [file DataSheet1.PDF]

Table S1. Sensitivity of state-of-the-art analysis of SPMs using LC-MS/MS of exemplary methods.

Shown is the lower limit of quantification of the instrument (LLOQ on column and the corresponding concentration) and the injection volume. Moreover, the effective LLOQ for liquid biological samples in pg/ml is shown, as well as the sample preparation technique, the initial sample volume, the reconstituted volume and the %-injected. All methods use electrospray ionization with triple-quadrupole analyzer listed either from Agilent (6470), Thermo (TSQ Quantum), Waters (Xeno TQS) or SCIEX (6500, 5500, 4000). Finally, the method used for definition of the LLOQ during method validation is given. Please note this table serves as orientation for the concentration range of the LLOQ (summarizing all covered SPM and exemplary LXA_4 and RvD2) and is not a comprehensive review about all available methods for oxylipin quantification.

Laboratory	Reference	SPMs covered (AA, EPA, DHA)	Instrument LLOQ		Injection volume	**LLOQ plasma/serum/ fluid**	Sample preparation	Initial volume	reconstituted volume (% injected sample)	Instrument	LLOQ definition	Comment
			pg on column	pg/ml vial	µl	**pg/ml**		µl	µl (%)			

**Dalli**	(Colas et al., 2014)	5S,15S-diHEPE, LXA_4<br><br>15R-LXA_4, LXB_4, RvE1, RvE2, RvE3, LXA_5, LXB_5, RvD1, 17*R*-RvD1, RvD2, RvD3, 17*R*-RvD3, RvD5, RvD6, PD1, 17*R*-PD1, 15*E*-PD1, 10*R*-15*E*-PD1, PDx, 22-OH-PD1, 22-COOH-PD1, MaR1, 12E-MaR1, 7S-12E-MaR1, 7S,14S-diHDHA, 4S,14S-diHDHA, 14S,21-diHDHA	0.05-0.22*,#,<br><br>d_5-LXA_4 0.05<br><br>d_5-RvD2<br><br>0.09	1.3-5.5,<br><br>LXA_4 1.3<br><br>RvD2 2.3	3)	0.05-0.22,<br><br>d_5-LXA_4 0.05<br><br>d_5-RvD2<br><br>0.09	SPE	1000	3)	6500	matching RT, min. 6 diagnostic ions

**Dalli**	(Gomez et al., 2020)<br>(Koenis et al., 2021)<br><br>**10.21203/rs.3.pex-1147/v1**	LXA$_4$, LXB$_4$, 15R-LXA$_4$, 15-LXB$_4$, RvE1, RvE2, RvE3, RvD1, RvD2, RvD3, RvD4, RvD5, RvD6, 17R-RvD1, 17R-RvD3, MaR1, MaR2, 4S,14S-diHDHA, 7S,14S-diHDHA, 22-OH-MaR1, 14-oxo-MaR1, PD1, 17R-PD1, 10S,17S-diHDHA, 22-OH-PD1	0.05-5.00,<br><br>LXA$_4$ 0.1<br><br>RvD2 0.1**, #	1.4-142,<br><br>LXA$_4$ 2.9<br><br>RvD2 2.9	35	0.05-5.00,<br><br>LXA$_4$ 0.1<br><br>RvD2 0.1	SPE	1000	40	5500		matching RT, ≥ 4 data points, > 2000 counts, 6 diagnostic ions, min. 1 backbone fragment
**Dennis**	(Dumlao et al., 2011)<br><br>(Deems et al., 2007)<br><br>(Quehenberger et al., 2011)	LXA$_4$, 6S-LXA$_4$,15R-LXA$_4$, LxA$_5$, LXB$_4$, RvE1, RvD1, PD1, 15E-PD1, 10S,17SdiHDHA	1*<br><br>LXA$_4$ 1	25	40	2.8	SPE	900	100 (20)	4000	S/N ≥ 3 (n=3) (LOD)	

**Geisslinger**	(Toewe et al., 2018)	LXA$_4$, 15R-LXA$_4$, 6R-LXA$_4$, LXB$_4$, RvD1, RvD2, 17R-RvD1, PDx, PD1, dinor-PD1, tetranor-PD1, LXA$_5$, MaR1	1-2,<br>LXA$_4$ 2<br>RvD2 2	100-200,<br>LXA$_4$ 200<br>RvD2 200	10	25-50,<br>LXA$_4$ 50<br>RvD2 50	SPE	200	50 (20)	5500	S/N ≥ 10, ± 20% accuracy and precision	Chiral chromatography
**Giera**	(Jónasdóttir et al., 2015)	LXA$_4$, 6S-LXA$_4$, LXB$_4$, RvE1, RvE2,18S-RvE3, 18R-RvE3, RvD1, RvD2, 10,17-diHDHA, MaR1	0.5<br>LXA$_4$ 0.5<br>RvD2 0.5	25<br>LXA$_4$ 25<br>RvD2 25	20	200	Protein precipitation with MeOH (96 well plate)	40	320 (6.3)	6500	S/N > 10	
**Giera**	(Jonasdottir et al., 2017)<br>(Giera et al., 2012)	LXA$_4$, 15R-LXA$_4$, LXB$_4$, RvE1, RvE2 18R-RvE3, 18S-RvE3, RvD1, 17R-RvD1, RvD2, 10S,17S-diHDHA, MaR1, 7S-MaR1	0.4<br>LXA$_4$ 0.4	10^{+}<br>LXA$_4$ 10<br>RvD2 no calibration	40	Synovial fluid<br>12	SPE	Synovial fluid<br>125	150 (27)	6500	S/N > 10	
**Hammock**	(Yang et al., 2009)	LXA$_4$	0.21	21	10	8	SPE	250	100	4000	S/N ≥ 10	

**Hersberger**	(Hartling et al., 2021)	RvE1, LXB_4, RvD2, RvD3, RvD1, 15R-LXA_4, 17R-RvD1, 6S-LXA_4, RvD4, PDx, PD1, RvD5, MaR1, MaR2,	0.002-0.063,<br><br>LXA_4 0.008<br><br>RvD2 0.002	0.2-6.3,<br><br>LXA_4 0.8<br><br>RvD2 0.2	10	0.4-12.5,<br><br>d_5-LXA_4 0.4<br><br>d_5-RvD2 3.2 [#]	SPE	200	50 (20)	6500+	S/N > 10	Use of alkaline mobile phase
**Mori**	(Mas et al., 2012)	RvD1, 17R-RvD1, RvD2, 10S17S-diHDHA, PD1	6	250	not provided	25	SPE	1000	100	TSQ Quantum	S/N ≥ 10	No individual values provided
**Newmann**	(Pedersen et al., 2021)	LXA_4, LXB_4, RvD1, RvD2, PDX, MaR1	0.2-1,<br><br>LXA_4 0.4<br><br>RvD2 0.8	40-199<br><br>LXA_4 80<br><br>RvD2 159	5	201-995<br><br>LXA_4 398<br><br>RvD2 794	Protein precipitation with ACN/MeOH (96 well plate)	50	250 (2)	6500	$3 \times t_{n-1,0.95} \times$ STD [§]	
**Nicholson**	(Wolfer et al., 2015)	LXA_4, LXB_4, RvD1, RvD2, 10S,17S-diHDHA	0.05-5,<br><br>LXA_4 1.3<br><br>RvD2 0.5	10-1000,<br><br>LXA_4 260<br><br>RvD2 100	5	12-1200,<br><br>LXA_4 312<br><br>RvD2 120	SPE	100	120 (4)	Xeno TQS	S/N > 5, intraday RSD < 20% (n=4), accuracy ±20%	

**Ramsden**	(Yuan et al., 2018)	LXA_4, LXB_4, RvD1, RvD2, RvD3, RvD4, MaR1, PDX,	1-5,<br>LXA_4 2<br>RvD2 5	100-500,<br>LXA_4 200<br>RvD2 500	10	20-100,<br>LXA_4 40<br>RvD2 100	SPE	200	40 (25)	5500	S/N > 5, intraday RSD < 20% (n=4), accuracy ±30%
**Schebb**	(Kutzner et al., 2019)	LXA_4, 15*R*-LXA_4, 6*S*-LXA_4, LXB_4, LXA_5, RvE1, RvE2, 18*S*-RvE3, 18*R*-RvE3, RvD1, 17*R*-RvD1, RvD2, RvD3, RvD5, MaR1,7*S*-MaR1, NPD1, PDx	0.6-3.6,<br>LXA_4 0.6<br>RvD2 1.4	61-360,<br>LXA_4 61<br>RvD2 141	10	6-36,<br>LXA_4 6<br>RvD2 14	SPE	500	50 (20)	6500	S/N ≥ 5,<br>± 20%<br>accuracy
**Werz**	(Werner et al., 2019)<br>(Werner et al., 2020)	LXA4, RvE3, RvD2, RvD4, RvD5, PD1, 17R-PD1, 10S,17S-diHDHA, MaR1	0.195-1.56,<br>LXA_4 0.195<br>RvD2 1.56*	19.5-156,<br>LXA_4 19.5<br>RvD2 156*	10	1-8,<br>LXA_4 1<br>RvD2 8*	SPE	Supernatant of cell incubations 2000	100 (10)	5500	S/N > 3, > 5 data points (LOD)

**Zhu**	(Wang et al., 2020)	LXA_4, LXB_4, RvE1 RvD1, RvD2, RvD3, RvD4, RvD5, MaR1, PD1,	0.18-4.5, LXA_4 0.18 RvD2 0.9	1.8-45, LXA_4 1.8 RvD2 9	100	5.4-135 LXA_4 5.4 RvD2 27	online-SPE	50	150 (67)	6470	S/N > 7

* only LLOD is given in the publication

** not specified between LLOD/LLOQ

[#] determined in plasma matrix

[+] lowest calibration level injected

[§] LLOD/LLOQ was determined based on a significant change (one-tailed t-test) in the sensitivity between successive calibration standards using the standard deviation (STD) of the concentration level significantly different than the preceding concentration level and the t-distribution (t-value: one-tailed, 95% confidence)

[1]calculated based on given parameters (blue)

[2]no information provided assumed based on common/given parameters (red)

[3]no information provided highest volume assumed
